# Genomic insights from whole genome sequencing of four clonal outbreak *Campylobacter jejuni* assessed within the global *C. jejuni* population

**DOI:** 10.1186/s12864-016-3340-8

**Published:** 2016-12-03

**Authors:** Clifford G. Clark, Chrystal Berry, Matthew Walker, Aaron Petkau, Dillon O. R. Barker, Cai Guan, Aleisha Reimer, Eduardo N. Taboada

**Affiliations:** 1 Division of Enteric Diseases, National Microbiology Laboratory, Public Health Agency of Canada, Canadian Science Centre for Human and Animal Health, 1015 Arlington Street, Winnipeg, MB R3E 3R2 Canada; 2 Bioinformatics Core Facility, National Microbiology Laboratory, Public Health Agency of Canada, Winnipeg, MB R3E 3R2 Canada; 3 Division of Enteric Diseases, National Microbiology Laboratory, Public Health Agency of Canada, Lethbridge, AB T1J 3Z4 Canada; 4 Department of Biological Sciences, University of Lethbridge, Lethbridge, AB Canada

**Keywords:** *Campylobacter*, Whole genome sequence, Phylogenetic analysis, Core genome MLST, Genomic inversions, Chemotaxis proteins, Transducer-like proteins

## Abstract

**Background:**

Whole genome sequencing (WGS) is useful for determining clusters of human cases, investigating outbreaks, and defining the population genetics of bacteria. It also provides information about other aspects of bacterial biology, including classical typing results, virulence, and adaptive strategies of the organism. Cell culture invasion and protein expression patterns of four related multilocus sequence type 21 (ST21) *C. jejuni* isolates from a significant Canadian water-borne outbreak were previously associated with the presence of a CJIE1 prophage. Whole genome sequencing was used to examine the genetic diversity among these isolates and confirm that previous observations could be attributed to differential prophage carriage. Moreover, we sought to determine the presence of genome sequences that could be used as surrogate markers to delineate outbreak-associated isolates.

**Results:**

Differential carriage of the CJIE1 prophage was identified as the major genetic difference among the four outbreak isolates. High quality single-nucleotide variant (hqSNV) and core genome multilocus sequence typing (cgMLST) clustered these isolates within expanded datasets consisting of additional *C. jejuni* strains. The number and location of homopolymeric tract regions was identical in all four outbreak isolates but differed from all other *C. jejuni* examined. Comparative genomics and PCR amplification enabled the identification of large chromosomal inversions of approximately 93 kb and 388 kb within the outbreak isolates associated with transducer-like proteins containing long nucleotide repeat sequences. The 93-kb inversion was characteristic of the outbreak-associated isolates, and the gene content of this inverted region displayed high synteny with the reference strain.

**Conclusions:**

The four outbreak isolates were clonally derived and differed mainly in the presence of the CJIE1 prophage, validating earlier findings linking the prophage to phenotypic differences in virulence assays and protein expression. The identification of large, genetically syntenous chromosomal inversions in the genomes of outbreak-associated isolates provided a unique method for discriminating outbreak isolates from the background population. Transducer-like proteins appear to be associated with the chromosomal inversions. CgMLST and hqSNV analysis also effectively delineated the outbreak isolates within the larger *C. jejuni* population structure.

**Electronic supplementary material:**

The online version of this article (doi:10.1186/s12864-016-3340-8) contains supplementary material, which is available to authorized users.

## Background

*Campylobacter jejuni* is the predominant bacterial species causing human enteric disease globally [1–5]. In the United States the reported number of human clinical cases is second only to *Salmonella*, and the incidence of disease attributed to *C. jejuni* is increasing in many countries [2]. This organism can be recovered from many animals, including cattle, pigs, chickens, wild birds, flies, and protozoa, from retail foods, especially chicken, and from the environment [4, 6, 7]. Despite the observation that many animals may serve as hosts for *C. jejuni*, poultry is thought to be the predominant source of human infections [4].

Surveillance for *C. jejuni* has been accomplished via DNA fingerprinting or molecular typing methods of variable discriminatory power, including pulsed-field gel electrophoresis (PFGE), multilocus sequence typing (MLST), ribosomal MLST (rMLST), flagellin short variable region (flaSVR) sequencing, and *porA* gene (major outer membrane protein, MOMP) sequencing [8, 9]. Using these methods, *C. jejuni* outbreak detection occurs relatively infrequently compared to detection of outbreaks caused by other enteric bacteria [10]. Water, milk, and chicken products are the most frequently reported sources of *C. jejuni* outbreaks [4, 10], with the majority of human cases assumed to be sporadic. Much of what we know about the passage of bacterial pathogens through the food chain and their interaction with human populations comes from characterization of foodborne-outbreak events [10]. The use of newer technologies and higher resolution methods like next-generation whole genome sequencing (WGS) provides more robust outbreak detection and characterization. In addition, comparative genomic methods can be used to investigate the biological and pathogenic mechanisms contributing to bacterial interactions with their environment, including their propagation and survival strategies as bacteria navigate the food chain to cause human clinical illness.

WGS is rapidly becoming a primary analytic method for bacterial phylogenetic studies, detection of bacterial pathogens in clinical laboratories, and outbreak detection and analysis [11–14]. However, in specific instances it may be more economically efficient to perform WGS on a smaller number of isolates representative of a larger population, such as when initiating studies aimed at the development of high-throughput, low-cost molecular subtyping assays for rapid or large-scale screening [9, 15]. Phylogenetic studies based on high-quality single nucleotide variant (SNV) distances and overall gene content have to date supported the current understanding that *C. jejuni* comprises a highly diverse group of organisms [3, 16]. Furthermore, WGS has been used to characterize isolates associated with milk- and water-borne *C. jejuni* outbreaks in Finland [17, 18] and to retrospectively identify case clusters from groups of isolates that were initially presumed to be associated with sporadic infections [19]. Genomic methods such as ribosomal MLST (rMLST), core genome MLST (cgMLST), and whole genome MLST (wgMLST) are complementary methods to extant, pre-genomic era typing schemes. Each of these newer genomic methods provides incrementally greater resolution, discriminatory power, and insight into *C. jejuni* populations while retaining the capability of accurately detecting case clusters [20]. However, the utility of WGS is not limited to phylogenetic analysis, and can provide clues about the virulence of an organism, its niche adaptation, its chromosomal structure, and other aspects of its biology [21–24]. We were therefore interested in undertaking a comprehensive analysis of isolates associated with the largest recorded Canadian *Campylobacter* outbreak, reasoning that valuable insights into the *Campylobacter* genome could be obtained through a robust analysis of whole genome sequences.

Four closely related *C. jejuni* isolates from a significant Canadian waterborne outbreak in 2000 [25] were subjected to WGS. These isolates were epidemiologically linked to the outbreak, previously typed as Walkerton outbreak strain 1 [26], and had common HS (heat-stable) and HL (heat labile) serotypes, biotype, MLST sequence type, and fla-SVR type. The PFGE restriction patterns varied according to the presence and location of the CJIE1 prophage within the bacterial chromosome [27]. Our previous investigations into the role of CJIE1 indicated that carriage of this prophage is linked to differences in bacterial phenotype [28] and protein expression [29]. Three isolates carrying the CJIE1 prophage exhibited increased adherence and invasion in cell culture compared with an isolate lacking the prophage [28]. Furthermore, there were differences in protein expression levels in the three isolates carrying the prophage compared with the isolate lacking the prophage [29]. These results suggested the CJIE1 prophage may affect diverse aspects of the biology of the organism. Previous, unpublished DNA-microarray data suggested that the isolates were genetically very similar except for the carriage of the CJIE1 prophage. However, it remained unknown if there were additional genetic differences other than those revealed using the earlier typing methods and DNA microarray technology.

Our first goal was to investigate the total genetic content among the isolates to determine if the isolates were 1) clonal, 2) differed mainly by the presence or absence of the CJIE1 prophage, and 3) harboured genetic differences with potential biological relevance other than the CJIE1 prophage. A second goal was to determine whether a thorough comparative genome analysis would provide additional insight into the population structure, genome plasticity, and virulence potential of the outbreak isolates. We determined that the genomic location and number of homopolymeric tracts within the four isolates and several other strains with complete, finished WGS suggested that the location and number of homopolymeric tracts may be useful to inform strain identity, relatedness, or clonal descent when assessing bacterial isolates that appear to be clonally or epidemiologically related. Comparative analysis also revealed the presence of a large, genetically syntenous chromosomal inversion, indicating structural heterogeneity of the chromosome and suggesting that the inversion may either have a biologically relevant phenotype or could be used as a marker specific for the outbreak isolates. Together, these results demonstrate the ability of WGS-based analysis to provide a great deal of in-depth, disparate, yet highly-valuable information about the organism under study.

## Results

### The major genomic difference among outbreak isolates is the presence of CJIE1 prophage

**Fig. 1:**
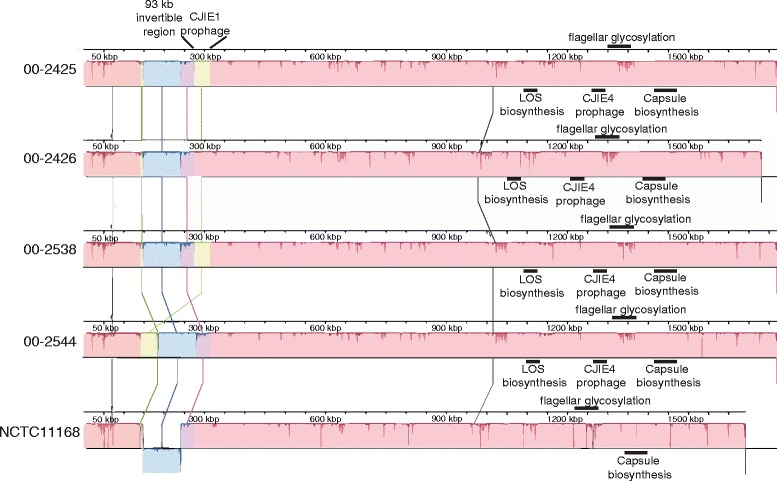
Alignment of the four *C. jejuni* outbreak isolates and reference strain NCTC11168 = ATCC 700819. The locations of the 93 kb invertible region and the CJIE1 prophage are shown. Approximate locations of CJIE4 prophages, capsule biosynthesis, flagellar glycosylation, and LOS biosynthesis regions are also shown. Note that the LOS biosynthesis region as shown also contains the general glycosylation region. The figure was prepared using Progressive Mauve (Mauve 20150226 build 10 [61])

**Fig. 2:**
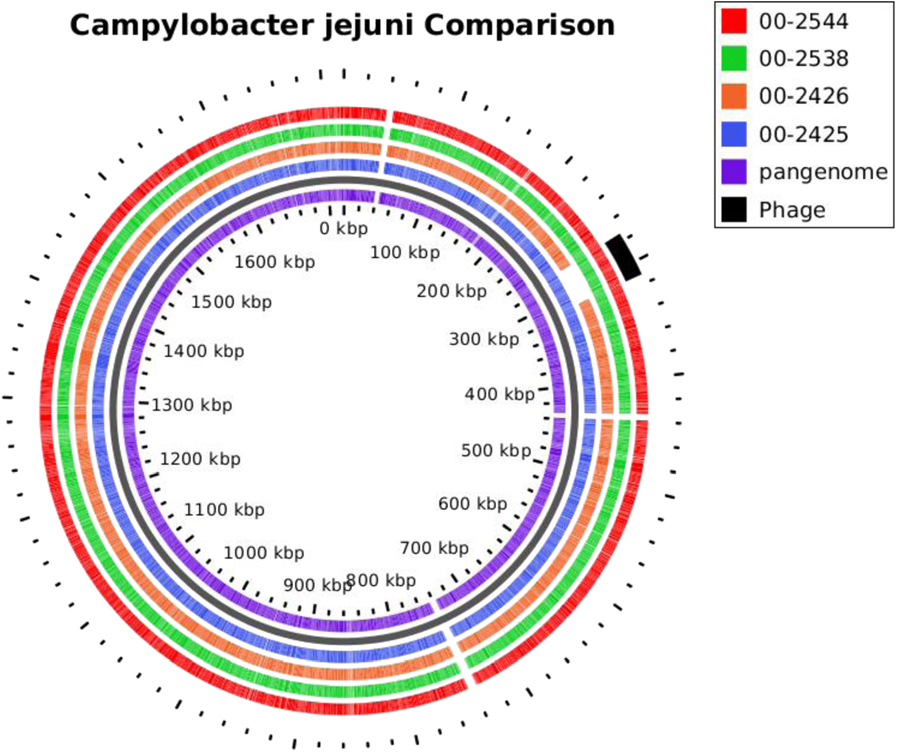
Blast atlas of whole genome sequences of *C. jejuni* isolates 00–2425, 00–2426, 00–2538, and 00–2544. The Blast atlas was prepared using GView Server [56]

Draft genomes of the four *C. jejuni* outbreak-associated isolates were obtained by paired-end sequencing on an Illumina MiSeq platform. Complete, closed, and finished genomes were obtained using a combination of read mapping to a reference genome, NCTC11168 = ATCC 700819, and bridging of contig gaps by Sanger sequencing (Fig. 1). The genome sizes of isolates 00–2425 (1,718,982 bases), 00–2538 (1,719,369 bases), and 00–2544 (1,719,532 bases) are very similar, differing by a maximum of 550 bases. In contrast, the genome size of isolate 00–2426 was 1,680,813 bases, a difference of between 38,169 and 38,719 bases from the previous three genomes. This difference is very close to the estimated size of prophage CJIE1 in *C. jejuni* isolate 00–2425 of approximately 38,000 bases. Both isolates 00–2425 and 00–2538 were annotated as having 1744 CDSs, compared with 1792 CDSs for isolate 00–2544 and 1686 CDSs for isolate 00–2426. DNA sequence alignments revealed that the four isolates were genetically syntenic with nearly identical gene content in all four strains apart from the CJIE1 prophage (Figs. 1 and 2), the presence of four tRNA loci (tRNA-Asp, tRNA-Val, tRNA-Glu, tRNA-Lys) near the *aas* gene only in isolate 00–2426 (data not shown), and the different chromosomal insertion site of CJIE1 in isolate 00–2544 (Fig. 1). This latter observation validated an earlier observation demonstrating chromosomal heterogeneity of the CJIE1 prophage in an otherwise clonal population of isolates [27].

Whole genome sequencing also revealed a 46,902 bp plasmid harboured by one isolate, *C. jejuni* 00–2544, showing high homology to pTet plasmids in the NCBI repository but varying due to the insertion of a transposable element comprised of the IS605 transposase (OrfB family) and integrase-resolvase (OrfA) genes adjacent to a gene encoding aminoglycoside 3'-phosphotransferase a short distance away from the plasmid-encoded *tet*(O).

### Phylogenetic analysis indicates that the four outbreak isolates are clonally related

**Fig. 3:**
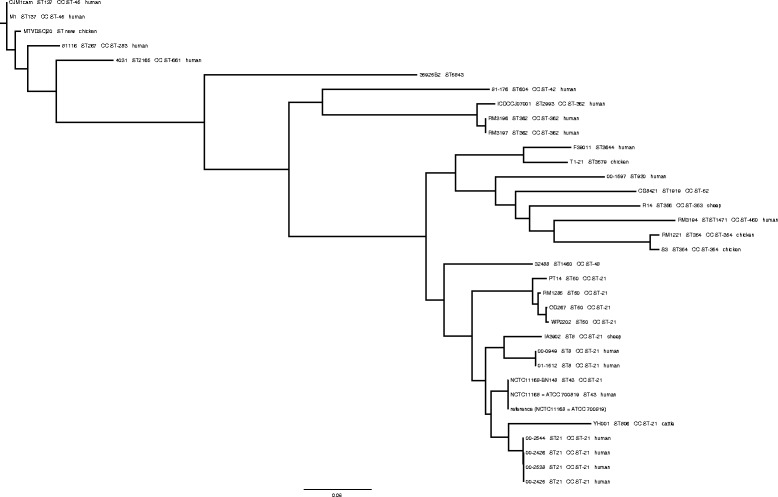
Phylogenetic tree of *C. jejuni* sequenced in this study compared with publicly available *C. jejuni*. The tree was constructed using hqSNV analysis as outlined in the Methods section

High quality SNVs (hqSNVs; Table 1) were identified and interrogated as outlined in the Methods section. Whole genome sequence alignments of the outbreak isolates to the reference strain, NCTC11168 = ATCC 700819, were performed. NCTC11168 = ATCC 700819 was selected as the reference for hqSNV analysis based on a Neighbor-Joining analysis using 31 publicly-available complete *C. jejuni* genomes that revealed this strain was a very close genetic neighbour to the outbreak isolates (Additional file 1: Figure S1). The core phylogenetic analysis revealed that, overall, the outbreak isolates differed by a total of 15 SNVs from one another, and in both the core genome and whole genome phylogenetic trees the outbreak isolates formed a distinct group within the overall topology of *C. jejuni* isolates (Fig. 3, Additional file 1: Figure S1). SNV analysis indicated they were most closely related to other MLST clonal complex (CC) ST-21 strains, especially YH001, which is sequence type (ST) 806, and the two NCTC11168 strains, which are ST43.

**Table 1:** High quality core SNVs in outbreak isolates using NCTC11168 = ATCCC 700819 as the reference

Position in NCTC11168	NCTC 11168	00– 2425	00–2426	00–2538	00–2544	Protein	Locus in NCTC11168
192795	G	A	A	A	G	amidophosphoribosyltransferase	Cj0196c
226072	C	A	A	A	C	intergenic	
231489	G	A	A	A	G	MFS transport protein	Cj0250c
492104	T	T	T	T	C	periplasmic protein	Cj0530
524447	C	C	T	C	C	periplasmic protein	Cj0561c
937334	C	C	C	C	T	MtaB	Cj1006c
959966	G	G	G	G	A	DNA gyrase subunit A	Cj1027c
1031608	T	T	G	T	T	serine/threonine transporter SstT	Cj1097
1137283	C	C	C	C	T	intergenic	
1173179	T	T	T	T	C	hypothetical protein	Cj1245c
1181839	G	A	A	A	G	LPS assembly protein	Cj1252
1189659	A	A	A	A	G	major outer membrane protein	Cj1259
1298000	A	A	A	A	G	secreted serine protease	Cj1365c
1515973	A	A	A	A	C	D-lactate dehydrogenase	Cj1585c
1630514	C	C	C	C	T	2-isopropylmalate synthase	Cj1719c

A hqSNV at position 1,035,018 in isolate 00–2426 (NCTC11168 position 1,031,608 in Table 1) results in the locus encoding the threonine/serine transporter SstT becoming a pseudogene. As discussed earlier [29] the NCTC11168 annotation and available data indicates that this protein is a serine transporter without any additional function and, as such, would not be expected to impart global regulatory effects. Of the 15 hqSNVs detected (Table 1), 13 were present only in isolate 00–2544 and two were found only in isolate 00–2426. In addition to the SNVs identified using the hqSNV core genome analysis, whole genome sequence using NUCmer (see Methods, [30, 31]) revealed several additional SNVs (Additional file 2: Table S1). Many of these were unique to isolate 00–2544, and were located in two genes encoding motility functions; flagellin A and the motility accessory factor protein homologous to Cj1341c. We hypothesize that these are allelic differences arising from homologous recombination. These data provide further evidence that isolate 00–2544 is not as closely related as the other three outbreak isolates. The analysis also revealed a SNV at position 682,886 in isolate 00–2425 (position 644,332 in isolate 00–2426) that would result in the phosphate acetyltransferase protein product being a pseudogene in isolate 00–2426. However, this position was located within a homopolymeric tract of T residues. Homopolymeric tracts are intrinsically repetitive and are therefore omitted from SNV analysis. The full-length phosphate acetyltransferase protein may be expressed due to rapid changes caused by homopolymeric tract length variability.

**Fig. 4:**
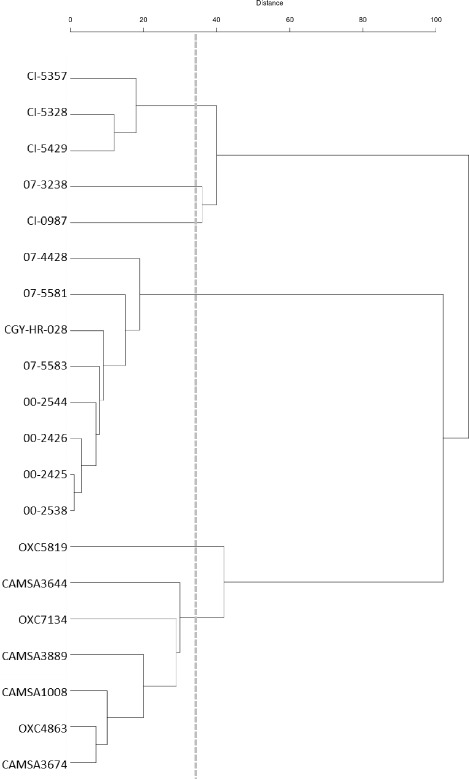
cgMLST tree showing phylogenetic relationships of the four outbreak isolates compared with other ST21 isolates. The tree was created using isolates within one of 21 clades (Clade 12) arising from a larger analysis of ST21 strains (unpublished data). Clade 12 contains *C. jejuni* strains most closely related to the four isolates (00–2425, 00–2426, 00–2538, 00–2544) sequenced for this work

We further subjected the four isolates to cgMLST analysis using a schema based on 732 core genes (unpublished data). Because the four outbreak isolates belong to ST21, we included additional genomes belonging to this sequence type obtained from the Bacterial Isolate Genome Sequence Database (BIGSdb; *n* = 181) [32] or sequenced by our group (*n* = 9) to provide greater context to the hqSNV results. The cgMLST results (Fig. 4) supported our phylogenetic findings and confirmed that the outbreak isolates were genetically highly similar. Core genome analysis revealed that ST21 represents a heterogeneous population of isolates comprised of distinct clusters with limited intra-cluster variability and high levels of inter-cluster variability (data not shown). Whereas the average number of variant loci between pairs of genomes was 172.1, the four outbreak isolates clustered with four additional Canadian isolates with an average of 9.39 variant loci. Within this cluster, isolates 00–2425, 00–2426 and 00–2538 showed the least amount of genetic variation, differing at an average of 2.0 loci. With an average of 5.6 variant loci, isolate 00–2444 showed slightly higher divergence with respect to the three other outbreak isolates. Four additional *C. jejuni* isolates were in the same immediate cluster as the Walkerton outbreak isolates 00–2425, 00–2426, 00–2538, and 00–2544 (Fig. 4). Three were isolated in 2007 from Ontario, while the fourth was isolated in 2006 from Alberta, indicating that this clade was both stable in time and geographically widespread. This group of eight strains was part of a larger cluster, Clade 12, which in turn comprised only a very small branch on a dendrogram of a ST21 phylogeny with a total of 21 clades (unpublished data). In this context the four Walkerton isolates are very closely related to each other and likely to be clonally derived, consistent with epidemiological data from the outbreak.

### Homopolymeric tracts are useful for assessing relationships among isolates

Because the number and location of homopolymeric tracts is known to vary among different *C. jejuni* isolates [33], we identified the locations of polyG/polyC tracts more than seven bases long in the four isolates sequenced for this study by searching DNA sequences in GenBank files for strings of Gs or Cs of eight nucleotides or longer and associating the position with the location of genes (Table 2).

**Table 2:** Consensus homopolymeric tracts in genomes of the four sequenced *C jejuni* isolates

Homolog in NCTC11168	Length of homopolymeric tract			
	00–2425	00–2426	00–2538	00–2544
115 bp upstream of L-asparaginase start codon (Cj0029)	10 C	10 C	10 C	10 C
Cj0045c iron-binding protein	**10 C**	**10 C**	**10 C**	**10 C**
Cj0170 methyltransferase	**8 C**	10 C	9 C	10 C
Between Cj0564 and Cj0565	10 G	11 G	11 G	10 G
Cj0617 carbonic anhydrase	9 G	**10 G**	**10 G**	9 G
Cj0628 lipoprotein	9 G	9 G	9 G	9 G
Cj0676 *kdpA* potassium-transporting ATPase A subunit	**10 G**	9 G	**10 G**	8 G
Cj0685c invasion protein CipA	**9 G**	8 G	**9 G**	**9 G**
Between Cj0742 (membrane protein) and 16S rRNA locus	10 C	10 C	10 C	10 C
Cj1051c *cjeI* restriction modification enzyme	**9 C**	10 C	**9 C**	**9 C**
Cj1139c *wlaN β*-1,3-galactosyltransferase	**8 C**	**8 C**	**8 C**	9 C
Cj1145 hypothetical protein	8 C	**10 C**	9 C	9 C
Cj1295 aminopeptidase	**9 G**	**9 G**	8 G	10 G
Cj1296 AAC(3) family N-acetyltransferase	9 G	9 G	9 G	9 G
Cj1305c carbonic anhydrase	10 C	**9 C**	10 C	11 C
Cj1306c carbonic anhydrase	**9 C**	**9 C**	**9 C**	**9 C**
Cj1310 hypothetical protein	**9 C**	**9 C**	**9 C**	10 C
Cj1321 promoter region; 37 bp upstream of Cj1321 start	10 G	10 G	11 G	11 G
Cj1325 methyltransferase	10 G	10 G	**11 G**	10 G
Cj1335–1336 motility accessory factor, maf4	**9 G**	10 G	**9 G**	**9 G**
Cj1342 motility accessory factor, maf7	8 C	**9 C**	**9 C**	**9 C**
Cj1420c methyltransferase	10 C	10 C	10 C	10 C
Cj1421c sugar transferase	**9 C**	**9 C**	**9 C**	**9 C**
Cj1422 sugar transferase	**9 C**	**9 C**	**9 C**	**9 C**
Cj1426c methyltransferase	11 C	12 C	9 C	11 C
Cj1429 hypothetical protein	11 C	8 C	**10 C**	11 C
Cj1437 hypothetical protein	**9 C**	**9 C**	**9 C**	10 C

Homopolymeric tract lengths corresponding to expression of the full length protein are in bold font

All four outbreak isolates carried 27 homopolymeric tracts in the same genomic locations, though the number of nucleotides within some of these exhibited apparent strain-specific variation. Consistent with previous reports [33], both the number and locations of homopolymeric tracts were different in the eight other strains included for comparisons (Additional file 3: Table S2), further supporting the premise that the four Walkerton *C. jejuni* are closely related clonal variants more similar to one another than to other *C. jejuni* strains. However, these four isolates carried 27 of the 29 homopolymeric tracts present in NCTC11168, missing homopolymeric tracts only in the type IIS restriction/modification enzyme (Cj0031) and the sodium:sulfate symporter (Table 2; Additional file 3: Table S2). In contrast, only 17/26 *C. jejuni* YH001 homopolymeric tracts were in the same location in *C. jejuni* YH001 and the four outbreak isolates. YH001 is an isolate from beef liver [34] that appeared to have substantial differences from the outbreak isolates and NCTC11168 (both HS:2) in the capsular polysaccharide biosynthesis region, which was most similar to regions encoding HS:4 (data not shown). Methods that do not take into account hypervariable regions may overestimate isolate relatedness. This was confirmed in the Neighbor-Joining analysis of *C. jejuni* complete genomes discussed in the previous section (Additional file 1: Figure S1).

Eight of the homopolymeric tracts present in the four Walkerton isolates were also present in a majority of the eight whole genome-sequenced strains analyzed (Additional file 3: Table S2). Their locations were within homologs of proteins annotated in NCTC11168 as Cj0045c (iron-binding protein/bacteriohemerythrin), Cj0617 (carbonic anhydrase), Cj0685c (invasion protein, CipA), Cj1295 (aminopeptidase), Cj1310 (hypothetical protein), Cj1342 (maf7), between Cj0564 and Cj0565, or between Cj0742 and the 16S rRNA locus.

### Identification and characterization of a previously unidentified large chromosomal inversion in outbreak isolates

The process of genome closing and finishing enabled the identification of a large, genetically syntenous region present in the outbreak isolates that was in reverse orientation compared to the reference genome. This was revealed during gap closure using PCR primers consistent with the reported genome sequences of *C. jejuni* strains NCTC11168 and RM 1221, shown in Table 3. These primers were designed to bind outside repeat regions at the ends of the contigs that affected the assembly of the genome.

**Table 3:** Primers used to detect the 93- and 388-kb inversions and the pTet plasmid

Primer target	Primer location	Primer name	Primer sequence (5´-3´)
Inversion (93kb)	*cj0143c*	Cj-F1	ATGCTTGAGGTGCTATACTGACAC
Inversion (93kb)	*cj0145*	Cj-R1	CCTTATCCTTAAGCATAGCAGCAC
Inversion (93kb)	*cj0261c*	Cj-F2	ACCCCAGTTCCACATCCTATATC
Inversion (93kb)	*cj0263 (zupT)*	Cj-R2	TGGTAAATTGGCAAACTCACAG
Inversion (388 kb)	*cj0261c*	CjInv5F	ACCCCAGTTCCACATCC
Inversion (388 kb)	*cj1562–1563*	CjInv5R	AACCCATCGACTTCATTTG
Plasmid	*cpp50*	cpp50tetOF1	GAACTTTACTTGCACGGAATGGAG
Plasmid	*tetO*	cpp50tetOR1	GGCCTGGCGTATCTATAATGTTGA
Plasmid	*virB4*	virB4rcvapF1	ATCTAGCTCATCATCATCTTCTGC
Plasmid	*vapD*	virB4rcvapR1	CTCGTCTTTCATCTATTGGTTCTT

**Fig. 5:**
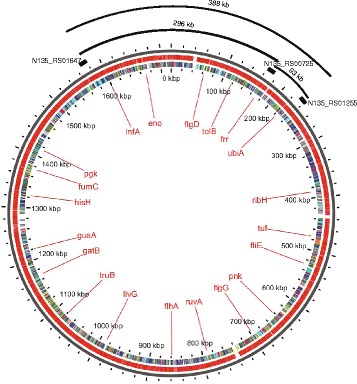
Schematic diagram of the 00–2425 genome with repeats and potential inversions. The image was obtained with the 00–2425 GenBank file using GView Server [56] and shows the locations of the repeat sequences associated with chemotaxis genes in the 00–2425 genome and genomic regions that may be inverted by recombination between these repeats. The approximate locations of the sequence repeats (*black boxes*) and the approximate size of the fragments that might be inverted are shown. The inside multicolored circle shows Clusters of Orthologous groups, while the red circle represents the 00–2425 genome

While use of the Cj-F1 and Cj-R1 or Cj-F2 and Cj-R2 primers pairs did not result in gap closure, PCR using the Cj-F1/Cj-F2 and Cj-R1/CjR2 primer combinations (Table 3) successfully amplified a ~2.5 kb product spanning the gaps between contigs. This indicates that within the genomes of the four isolates an approximately 93-kb section of the genome between genes homologous to *cj0143* (ABC transporter binding protein) and *cj0263* (ZupT) was inverted relative to NCTC11168 (see Fig. 5, Additional file 4: Figure S2, Additional file 5: Table S3) and several other annotated *C. jejuni* genomes within the NCBI database (data not shown). The NCTC11168 strain (HS:2, ST43) used in our laboratory as a reference strain for PCR determination of inversion sequence termini was found to harbour an ~92-kb chromosomal inversion that was extremely similar to the four sequenced outbreak isolates. This experimental finding was in contrast to the sequence annotation for NCTC11168 = ATCC 700819 in NCBI, which does not contain an annotated inversion. It is unclear if this was because our reference strain is a variant of NCTC11168 arising from repeated laboratory passage or if this is the result of an error with the annotated sequence in the NCBI repository. Analogs of the 93-kb inversion were not limited to HS:2 or ST21 isolates, though to date the inversions we have found all belong to CC ST-21 strains. An inversion with the same core gene content was detected in isolates 00–6200 (HS:4,13, ST806, CC ST-21; NCBI accession No. NZ_CP010307) and YH001 (HS:4, ST806, CC ST-21; NCBI accession No. NZ_CP010058.1).

The *C. jejuni* NCTC11168 original annotation (GenBank Accession No. AL111168) documents the existence of three large sequence repeats located in the loci *cj0144*, *cj0262c*, and *cj1564*, all of which were annotated as methyl-accepting chemotaxis proteins, later renamed transducer-like proteins (Tlps) [35]. Different Tlps have divergent N-termini, with the repeats located in DNA sequences encoding the C-termini. The repeats associated with *cj0144* and *cj0262* flank a region in NCTC11168 of 92,358 nt that shares 99% nt identity with the 93,129-kb inverted region in the four outbreak isolates (Additional file 4: Figure S2; Additional file 5: Table S3). Genome analysis revealed that each of the four isolates sequenced for this study (00–2425, 00–2426, 00–2538, 00–2544) also harbour large sequence repeats within genes analogous to those in NCTC11168. These repeats were in proteins annotated as a methyl-accepting chemotaxis protein (Tlp3), a ribose and galactose chemoreceptor protein (a novel Tlp, here designated Tlp12), and a methyl-accepting chemotaxis protein (a second copy of Tlp3); the first Tlp3 and Tlp12 flank the 93-kb invertible element in the outbreak isolates. The gene content within the invertible region may be maintained even though there may be changes in the adjacent methyl-accepting chemotaxis loci/Tlps. This warrants further investigation.

The repeat sequences associated with each of the three *tlp* loci displayed 100% sequence identity in all four outbreak isolates, although the different *tlp* genes harbour repeats of different lengths (Additional file 6: Figure S3). In the outbreak isolates the first repeat (going clockwise from the origin of replication, Fig. 5) was 1979 nt, the second 1011 nt, and the third 1970 nt (Additional file 6: Figure S3). We have designated the 1979 nt repeat unit “repeat 1”, the 1101 nt repeat unit “repeat 2” and third 1970 repeat unit “repeat 3”. Repeat 2 is in reverse orientation to the other two in the outbreak isolates, and all three repeats display high levels of nucleotide identity over variable sequence lengths: 1969/1970 (99%) between repeats 1 and 3; 1004/1011 (99%) between repeats 1 and 2; 994/1004 (99%) between repeats 2 and 3 (Additional file 7: Figure S4). The two *tlp3* genes exhibit identity over 1955/1956 nt using NCBI blastn, and the respective proteins are identical over 650/651 aa when aligned using NCBI blastp. Repeats 1 and 3 are both larger than the *tlp3* genes with which they are associated. We hypothesize that the high percentage of nucleotide identity between repeats is sufficient to support recombination events leading to the large chromosomal rearrangements and subsequent invertible region.

The corresponding repeat 1 in NCTC11168, associated with the locus *cj0144* encoding a Tlp2 protein, is 1079 nt; this repeat displays 99% identity over 1079 nt with the first repeat in all four sequenced outbreak isolates. Repeat 2 in NCTC11168, associated with locus *cj0262c* encoding a Tlp4 protein, is 1147 nt in length, and has 1000/1013 (99%) identity with the second repeat in the four outbreak isolates except for four indels. It is placed in reverse orientation relative to the first repeat in NCTC11168 similarly to the first two repeats in the four outbreak isolates. Finally, repeat 3 in NCTC11168, associated with locus *cj*1563c encoding Tlp3, is 1079 nt compared with 1970 nt for repeat 3 in the sequenced outbreak isolates. There is a 1951/1956 (99.7%) nt identity between the NCTC11168 and outbreak-isolate *tlp3* genes. Studies are underway to investigate the diversity and extent of these repeat units within an expanded *C. jejuni* population and will be the subject of a separate report.

The maintenance of two sequence repeats flanking an invertible element may indicate that the element harbours genes subject to selective pressure(s). This hypothesis is supported by the observation that following an inversion event genes on the leading strand will now be switched to the lagging strand with respect to chromosomal replication, effectively changing the gene dosage [21, 35]. Inspection of the predicted protein identities for genes within the 93 kb invertible cassette revealed almost identical gene content, with very few exceptions (Additional file 4: Figure S2, Additional file 5: Table S3). BLAST searches using nucleotide sequences of the 92-kb invertible region (including repeats) of NCTC11168 = ATCC700819 as the query sequence and the 93-kb invertible region of isolate 00–2425 as the subject sequence identified a region with of 92130/92207 (99%) identity with 23/92207 gaps (0%), an Expect value of 0.0, and a BLAST score of 1.659e + 05 bits (184022). Thus, despite the annotation differences, the gene/protein content of the two regions is highly similar. Included among the loci encoded in the invertible element are a number of membrane proteins, chemotaxis proteins, RNA-modifying enzymes, and a subset of iron acquisition proteins on the leading strand of the four *C. jejuni* isolates, some of which are proposed to have a functional role in environmental or niche adaptation. In contrast, the lagging strand (genes in complementary orientation) carried a large complement of transport proteins, including theTonB receptor and TonB transporter, ExbB and ExbD, among others.

### Outbreak isolates are characterized by a 93-kb chromosomal inversion but demonstrate variable pTet plasmid carriage

We were interested in determining whether the 93 kb inversion was a characteristic feature of isolates associated solely with the Walkerton outbreak. Not all isolates associated with the outbreak were previously typed by molecular typing methods or PFGE [26] though all were subjected to phage typing, HS typing, and HL typing [36]. Therefore, MALDI-TOF was used to verify the species (*jejuni* or *coli*) of selected isolates (data not shown), and MLST types were determined retrospectively for 95 additional *C. jejuni* isolates from the outbreak analysis. Isolates from humans or bovine manure were considered to be associated with the outbreak strains [26] if they were isolated during the outbreak investigation, considered epidemiologically outbreak-associated based on time and location, and belonged to CC ST-21. We were interested in identifying outbreak-strain markers for analysis of this particular outbreak or that could have been used to better characterize the *Campylobacter* population in that particular geographical area at that time.

PCR detection of the 93-kb inversion indicated that the inversion was a characteristic of outbreak strains since it was detected in 97% of the human isolates and 100% of the cattle isolates (Table 4), consistent with the clonal spread of the organism within the local cattle population and its subsequent introduction into humans through water [25]. The inversion was not prevalent in other CC ST-21 isolates tested, though the numbers were small (Table 4). The inversion was present at much lower frequencies in the relatively few non-Walkerton-outbreak isolates available and was detected in an isolate of strain NCTC11168 which has been utilized for years in our laboratory as a reference strain for different investigations. Together these results indicate that the 93-kb inversion was closely associated with the Walkerton *C. jejuni* isolates and could be used as a secondary, confirmatory trait of outbreak isolates. Although PCR detection of the inversion was highly effective and reliable as a tool in this instance, and could be rapidly used during an outbreak investigation, WGS-based analysis using the methods described in this paper were equally effective.

**Table 4:** Detection of a 93-kb inversion in isolates associated with the Walkerton outbreak investigation

Isolate characterization	Detection of 93 kb inversion using PCR primers			
	Total isolates	Non-inversion	Inversion	No PCR product
Walkerton human, CC ST-21	110	3 (3%)	107 (97%)	0
Walkerton human, not CC ST-21	14	14 (100%)	0	0
Walkerton bovine, CC ST-21	22	0	22 (100%)	0
Walkerton bovine, not CC ST-21	20	15 (75%)	1 (5%)	4 (20%)
Non-Walkerton human, CC ST-21	10	8 (80%)	1 (10%)	1 (10%)
Non-Walkerton human, not CC ST-21	4	3 (75%)	0	1 (25%)
Non-Walkerton animal, CC ST-21	5	3 (60%)	1 (20%)	1 (20%)
Non-Walkerton animal, not CC ST-21 (*C. coli*)	8	0	0	8 (100%)
Total	193	46	132	15

The discovery of a pTet plasmid homolog in a single outbreak isolate was accomplished using two sets of PCR primers (Table 3). Amplicons for both primer sets were detected in 15% of the human Walkerton CC ST-21 isolates, increasing to 64% detection among Walkerton CC ST-21 isolates from cattle manure (Table 5). The plasmid was detected at higher frequencies in non-Walkerton CC ST-21 isolates from humans and animals, as well as in *C. coli* (not CC ST-21) isolates from animals. It appeared that either the human population was less frequently infected with plasmid-containing strains from bovine feces or that the plasmid was readily lost upon human infection. In a minority of cases only one of the two amplicons was detected, suggesting some heterogeneity in the plasmid content or nucleotide changes within the primer binding sites. The plasmid was not a reliable marker for outbreak strains and appears to have been rapidly lost in the human population after infection with strains of bovine origin through contaminated water.

**Table 5:** Detection of two PCR products from the pTet plasmid in Walkerton outbreak investigation strains

Isolate characterization	Detection of virulence plasmid PCR products				
	Total isolates	Both PCR products	cpp50tetO	virB4rcvap	No plasmid
Walkerton human, CC ST-21	110	16 (15%)	0	3 (3%)	91 (83%)
Walkerton human, not CC ST-21	14	1 (7%)	2 (14%)	2 (14%)	9 (64%)
Walkerton bovine, CC ST-21	22	14 (64%)	0	3 (14%)	5 (23%)
Walkerton bovine, not CC ST-21	20	2 (10%)	0	1 (5%)	17 (85%)
Non-Walkerton human, CC ST-21	10	9 (90%)	0	1 (10%)	0
Non-Walkerton human, not CC ST-21	4	0	0	0	4 (100%)
Non-Walkerton animal, CC ST-21	5	2 (40%)	0	0	3 (60%)
Non-Walkerton animal, non CC ST-21	8	4 (50%)	0	0	4 (50%)
*C. coli*					
Total	193	48	2	10	133

Though data were limited, 7/10 of the non-Walkerton human CC ST-21 isolates with the complete pTet plasmid belonged to two PFGE types. Of these, five had PFGE patterns CASAI.0009-CAKNI.0032 and were from New Brunswick (2), Québec (2), and Ontario (1), while one each from Québec and New Brunswick had PFGE patterns CASAI.0026-CAKNI.0031. This suggests the pTet plasmid may have been circulating in a geographically delimited reservoir of related isolates.

### Identification of a 388 kb inverted region

We hypothesized that additional chromosomal inversions could have arisen in *C. jejuni* due to homologous recombination events mediated by the high nucleotide identity observed among the three repeat elements identified in the sequenced outbreak isolates (see Fig. 5). PCR analysis using primers designed to bridge the junctions of each of these potential inversions was performed on a subset of *C. jejuni* and *C. coli* isolates (see Methods, Strains and growth conditions), including isolates that did not harbour either the non-inverted sequence or the 93-kb inversion. Only one isolate (00–5949; HS:2, ST21, *fla*-SVR type 49) produced an ~3 kb amplicon using the cjinv5 primer set designed to specifically detect an inversion between gene homologs of *cj0261c* and *cj1562-1563* (Table 3). Sanger sequencing and BLAST analysis of this 3-kb product indicated that the amplified sequence was consistent with a ~388 kb inversion between the *tlp3* and *tlp12* genes present in the four sequenced outbreak isolates. A third possible inversion of 297 kb located between repeats 1 and 3 (in the two *tlp3* genes) was not detected in this study. The sizes of the 388-kb inversion and potential 296-kb inversion would each be increased by 38 kb in isolate 00–2544 due to the insertion of the CJIE1 prophage (see Fig. 1).

## Discussion and Conclusions

The four outbreak isolates characterized in this work had closed, finished genomes with almost identical gene content, with the exception of a deletion of the CJIE1 prophage, four additional tRNA loci in isolate 00–2426, and a hqSNV inactivating the threonine/serine transporter SstT. They also had the same homopolymeric tract content and location, minimal hqSNV variability, and limited cgMLST allelic variation, especially in the context of a larger population of ST21 strains. These data provided strong support for the conclusions presented in earlier work that the strain-specific differences in cell culture adherence and invasion assays [28] and protein expression [29] resulted from the presence or absence of the CJIE1 prophage. Though artifacts may arise from the use of SNV analysis with *C. jejuni* [16], we found that both SNV analysis and cgMLST delivered congruent results in that they clustered the four outbreak isolates closely together within the *C. jejuni* phylogenetic structure. Both hqSNV analysis and cgMLST demonstrated consistent differences between isolates that were due to allelic differences at a small number of loci, supporting the hypothesis that they were changing rapidly as part of a freely recombining population. Interpretation of whole genome sequence data relies on large datasets to allow conclusions about phylogenetic relationships to be drawn; a larger dataset was used for the cgMLST analysis, which enabled a much more detailed picture of phylogenetic relationships. Crucial phylogenetic information not available using hqSNV analysis or cgMLST was provided by comparing the complete genomes of WGS strains, which contain the accessory genome for each strain, using whole genome Neighbor-Joining phylogenetic methods.

### Homopolymeric tracts are useful for assessing relationships among isolates

The locations of homopolymeric tracts were identical in the four outbreak strains and variable from other strains assessed. These observations were consistent with the phylogenetic relationships among isolates determined in this work showing that the four Walkerton outbreak strain 1 isolates were related to, but distinct from, strain NCTC11168 (Fig. 3, Additional file 2: Figure S1). They also support our conclusions that 00–2425, 00–2426, 00–2538, and 00–2544 are genetically almost identical and are most certainly clonally derived, thus highlighting the value of using comparisons of homopolymeric tract locations as an adjunct method to confirm phylogenetic relationships within *C. jejuni*. While the homopolymeric tract content and location may provide supporting data regarding the relatedness of strains for outbreak analysis, it seems unlikely that these data will supplant cgMLST and SNV analysis for outbreak detection and analysis or investigations into population genetics of the organism.

Changes in homopolymeric tract length affect expression of the genes with which they are associated and their association with different loci in different strains suggests that these homopolymeric tracts contribute to the adaptation of strains through selection in different environments or niches [33]. When population data are available, each of the homopolymeric tract length variants detected is best described as a proportion or percentage of the total population [37–39], and even individual strains of *C. jejuni* are a heterogeneous population of organisms capable of generating multiple phenotypes through adaptation to external or environmental conditions [40]. However, due to the high rate of mutation causing nucleotide changes in homopolymeric tracts [33] SNVs arising from changes in polyG/polyC tract lengths are not useful for phylogenetic or molecular epidemiologic analysis and it may be of merit to consider SNV differences after removing them from the analysis [17], as well as adjusting the lengths of the homopolymeric tracts to encode the full length protein when submitting sequence data to the NCBI.

### Potential significance of large chromosomal inversions

Detection of large chromosomal inversions between chemotaxis proteins/Tlps containing large conserved repeats indicates that *C. jejuni* genomes may be more dynamic than previously appreciated and suggests that this property may have adaptive consequences. Chromosomal inversions may not be uncommon in *C. jejuni* and related organisms. In *Helicobacter pylori*, an organism closely related to *Campylobacter* spp., large chromosomal inversions were associated with insertion element IS605 (producing a 75 kb inversion) or inverted copies of repeat 7 (producing an 83 kb inversion) [41]. In *C. jejuni* strains, large genomic rearrangements induced by lytic bacteriophage predation resulted from recombination between two copies of the CJIE1 Mu-like bacteriophage [42]. One inversion characterized in this study was centered on the origin of replication and had measurable biological consequences that included increased survival in adverse ecological conditions. Inversions between *rrn* operons in the genomes of specialist *Salmonella enterica* serotypes Typhi, Paratyphi C, Gallinarum, and Pullorum have been detected at much higher rates than in the generalist *S*. Typhimurium in spite of similar frequencies of inversion, an observation attributed to decreased survival of *S*. Typhimurium due to selection pressures inherent to its generalist lifestyle [43]. *C. jejuni* associated with several sequence types, including ST21, have been described as generalists on the basis of phenotypic flexibility and high genetic microdiversity [44]. It is not clear whether this affects the frequency of chromosomal inversions in this organism, or whether there are chromosomal inversions mediated by *rrn* operons or other repeats detected in *C. jejuni* chromosomes. Inversions that do not maintain the genome balance, defined as the lengths of replichores between the origin and terminus of replication, are also subject to selective pressure [21]. The 93 kb inversion we detected would likely not affect the genome balance except in the case of strain 00–2544. Further investigations are necessary to determine whether the three repeats found in strains used in this study are restricted to only a subset of *C. jejuni* or are more generalized throughout the population, as well as whether the gene content of the 93-kb invertible region is relatively conserved or diverse in content. Too few data were obtained to enable determination of an estimate of inversion frequencies between the repeats. It has been suggested previously that repeats separated by sequence comprising more than 10% of the chromosome length are rare, indicating strong counter selection against very large inversions [45]. Higher rates of inversion would tend to homogenize a population distinguishable by other means, while lower rates may lead to increased temporal and geographic stability, thus enabling inversion status as a useful measure for population biology. This contention is supported by the results of experiments assessing the frequency of the 93 kb inversion in Walkerton outbreak isolates.

Differences in gene placement between the leading or lagging strand and differences in distance from the origin of replication that would result from inversion of the genomic segment(s), with possible concomitant gene dosage changes, may be associated with differences in gene and protein expression that would favor different lifestyles or environments. However, since the 93-kb inverted element is relatively small and close to the origin of replication there may not be sufficient difference in gene dosage to be of significance. This is a subject for future investigation.

Analysis of complete, finished whole genome sequences of four outbreak isolates revealed chromosomal inversions and provided insight into the capability of using changes in homopolymeric tract locations as an estimator of isolate relatedness. The examination of whole genome sequences for properties in addition to those that assess phylogenetic relatedness for resolving outbreaks provides additional valuable insights into the biology of the organism. By closing and finishing a representative subset of outbreak genomes, we have been able to gain insight into the dynamics of the *Campylobacter* genome, information that would not have been reliable or accurate using draft genomes alone. This analysis is anticipated to spur additional research into unravelling the intricacies of the *Campylobacter* genome and augment our existing knowledge of outbreak-relevant genomic markers.

## Methods

### Strains and growth conditions

The four isolates selected for whole genome sequencing and comparative genomic analysis were associated with the investigation into the spring 2000 *Campylobacter* and *E. coli* outbreak in Walkerton, Ontario, Canada, and have been described previously (see Background). Other isolates were included to determine the prevalence of a 93 kb inversion and a large 46,902 bp pTet plasmid in isolates associated with the Walkerton outbreak investigation. These isolates were all linked to the Walkerton outbreak or the subsequent investigation [26, 45] and included *C. jejuni* and *C. coli* isolates not previously characterized by PFGE or other molecular methods. Among the isolates used were 124 isolates from humans (110 outbreak types and 14 non-outbreak types) and 43 cattle feces isolates (23 outbreak types and 20 non-outbreak types) collected as part of the outbreak investigation. Additional *C. jejuni* non-outbreak isolates were analyzed to provide additional context for the outbreak [26]: 13 non-outbreak *C. jejuni* from five different Canadian provinces and one from Egypt obtained from 2000 to 2003 (11 with outbreak type CC ST-21 but different PFGE types than outbreak isolates); two non-outbreak *C. jejuni* from Ontario cattle isolated in 2000; one Ontario canine isolate from 2000 with CC ST-21 but different PFGE types than outbreak strains; two Louisiana chicken isolates from 1999 with CC ST-21. Eight *C. coli* isolates obtained during the outbreak investigation, two from humans and six from cattle stools were also used here. MLST was done as described previously [26].

*C. jejuni* isolates were maintained in either 20% skim milk or glycerol peptone water (25% v/v glycerol, 10 g/L neopeptone, 5 g/L NaCl) at −80 °C. For use, *C. jejuni* isolates with a low passage number were retrieved from storage at −80 °C, plated to Oxoid Mueller-Hinton agar (Oxoid Inc.) containing 10% sheep red blood cells (OMHA + blood), and grown for 48 – 72 h at 37 °C under a microaerobic atmosphere (5% O_2_, 10% CO_2_, 85% N_2_).

### PCR for determining the presence of the 93- and 388-kb genomic inversions and pTet plasmids

All PCR reactions were done using DNA extracted from bacteria using a Gentra Systems PUREGENE DNA Isolation kit (Qiagen) according to the instructions of the manufacturer. PCR reactions were run using reagents from FastStart Taq DNA Polymerase kits (Roche). PCR reactions using the Cj primer sets to assess the presence or absence of the ~93-kb and ~388-kb inversions (Table 1) each consisted of a 50 μl reaction mix at final MgCl_2_ concentrations of 2.0 mM, 0.2 mM of each dNTP, 0.2 μM of each primer, and 0.2 U of FastStart DNA polymerase. PCR reactions to detect the 93-kb inversion were run using Cj-F1 and Cj-F2 primers, while the non-inverted configuration was detected using the Cj-F1 and Cj-R1 primer set. Amplification for both the Cj-F1/Cj-R1 and CjF1/CjF2 sets of primers utilized the following cycles: 1 cycle of 95 °C for 2 min; 35 cycles of 94 °C for 30 s, 56 °C for 30 s, 68 °C for 5 min; 1 cycle for final extension at 72 °C for 7 min; 4 °C until samples were retrieved. The amplicon obtained using both the F1-R1 primers and F1-F2 primers was approximately 2.5 kb in size. Strain RM1221 was the positive control for the non-inverted configuration, NCTC11168 was the positive control for the inversion, and a tube or well containing water instead of DNA was a negative control to detect inappropriate amplification. For detection of the 388-kb inversion the Cjinv5 primers cycle conditions were: 1 cycle of 95 °C for 2 min; 35 cycles of 94 °C for 30 s, 47.8 °C for 30 s, 68 °C for 4 min; 1 cycle for final extension at 72 °C for 7 min; 4 °C until samples were retrieved. The Cjinv5 amplicon was approximately 2.5 kb. Other potential inversion ends were tested but no amplicons were obtained; the primers used were therefore not included here.

Two sets of primers were designed for detection of the pTet plasmid in *C. jejuni* isolates associated with the Walkerton outbreak investigation using the sequence of the plasmid obtained for *C. jejuni* strain 00–2544 (Table 1). Each 50 μl reaction mix contained final MgCl_2_ concentrations of 2.0 mM, 0.2 mM of each dNTP, 0.5 μM of each primer, and 0.2 U of FastStart DNA polymerase. Amplification cycles were the same as above for the primers to detect inverted or non-inverted genomic sequences, but the cpp50tetO primer set used an annealing temperature of 53 °C and an extension time of 45 s while the virB4rcvap primer set had an annealing temperature of 50 °C and a 2 min extension time. The product of the cpp50tetO amplification was 1018 bp; that of the virB4rcvap amplification was 1410 bp. These two primer sets amplified regions that were approximately 19 kb apart in the 46 kb plasmid. The positive control was DNA from 00-2544, the negative control was DNA from NCTC11168, and a water blank was included as a contamination control for each run.

All PCR reactions were performed using a Gene Amp 9700 thermocycler (Applied Biosystems). Visualization of PCR products was accomplished using submarine gel electrophoresis followed by staining with GelRed Nucleic Acid Stain (Cedarlane) or analysis using a Qiaxcel instrument (Qiagen).

### Genome sequencing, assembly, closure, and annotation

#### Sequencing

Genomic DNA was prepared from four selected Walkerton outbreak isolates cultured overnight at 42 °C on OMHA + blood using Epicentre Metagenomic DNA Isolation kits for Water (Illumina) according to the manufacturer’s instructions. Quantitation of DNA was accomplished using Qubit dsDNA BR assay kits (Life Technologies, Invitrogen). Sample libraries were prepared using MiSeq Nextera® XT DNA library preparation kit (Illumina). Whole genome sequencing was performed by 250 bp paired-end read sequencing on the Illumina MiSeq sequencer using MiSeq® Reagent Kit V2 and 500 cycles on the Illumina MiSeq platform to obtain an average genome coverage of 30–50×. Sequence reads were assembled into contigs using the SPAdes assembler (v3.0 [46]). Contigs smaller than 1-kb and with average genome coverage less than 15× were filtered and removed from the analysis. The remaining contigs were closed and finished using Staden gap v4.10 by read mapping to the reference genome, NCTC11168, and a combination of PCR and Sanger sequencing for gap closure. Fasta files for each genome were sent to Genomes (NCBI) for annotation using the NCBI prokaryotic annotation pipeline. Additional information for selected loci was then added manually.

### Core genome hqSNV analysis

A core genome phylogeny was constructed from high quality variants within the core genome using the read data for *C. jejuni* isolates 00–2425, 00–2426, 00–2538, 00–2544, and 29 related isolates from GenBank using the closed and finished genome of isolate 00–2425 as the reference. The Illumina MiSeq data for the isolates sequenced in this study were first concatenated into one fastq file per isolate containing both forward and reverse reads. Fasta files for closed, finished genomes of 29 additional isolates were downloaded from GenBank and converted to reads of 250 bp using custom Perl scripts (WombacShred [47]) to generate fastq files. The set of reads generated for each isolate was used for phylogenetic SNV analysis using the PHAC-NML high-quality single nucleotide variants (SNVPhyl) pipeline [48].

In summary, read data for each genome was mapped against a reference closed and finished genome (NCTC11168 = ATCC 700819) using SMALT (v. 0.7.5; Wellcome Trust Sanger Institute, Cambridge, UK). The SMALT parameters used were a smalt index kmer size of 13, a step size of 6, and a minimum alignment fraction of 0.5. Variants were called using FreeBayes (v. 0.9.20 [49, 50]) with the “--ploidy 1” parameter for haploid variant calling. For each VCF file created by FreeBayes, an in-house script was used to filter out complex variant calls with indels, split the remaining complex variant calls into single variant calls, and create new VCF files. The BCFtools component of the SAMtools package (v. 1.3 [51]) was used as a second variant caller to validate the variant calls made by FreeBayes. All variant and non-variant calls from FreeBayes and BCFtools were merged together and positions where variant calls were not in agreement between both variant callers and which did not have a minimum coverage of 10, minimum alternative ratio of 0.75, and minimum mean mapping quality of alternative alleles of 30 were excluded from the analysis. Additional filtering was performed to remove variant calls within repetitive regions (identified using MUMmer v3.23 [31] with a minimum percent identity of 90 and minimum length of 150 bp) and those within high SNV-density regions (identified with a sliding window of size 20 bp and minimum threshold of two SNVs within this window size) to be removed. All remaining variant calls were merged into a single meta-alignment file. The meta-alignment was used to generate a maximum likelihood phylogenetic tree with PhyML [52]. The dendrogram was depicted with FigTree v1.4 [53]. Metadata for isolates obtained from the NCBI repositories was obtained from the scientific literature. When necessary, MLST designations were obtained from WGS data using custom in-house scripts [54].

### Pan-genome BLAST Atlas

A pan-genome BLAST Atlas was created using GView [55] and GView Server [56]. The pan-genome was constructed iteratively by starting with the predicted chromosomal regions (CDS) in 00–2425 and concatenating unique regions among the other three genomes using MUMMer (v3.1) alignments. Next BLASTn was performed between the pan-genome and each of the other genomes. Regions on each genome reporting a BLAST hit above the threshold cutoff (80% identity, minimum HSP length of 100 bp, and expect value of (1e-10)) were considered a valid match and drawn on the pan-genome BLAST atlas.

### cgMLST analysis

A cgMLST scheme was developed as previously described [57] using allele definitions obtained from BIGSdb [32]. A dataset of genomes comprised of isolates from ST21, which included genomes obtained from BIGSdb (*n* = 181) and those sequenced by our group (*n* = 17), was analyzed by cgMLST. Analysis was performed using the Microbial In Silico Typer (MIST) software [58]. Briefly, MIST was used to query each genome for all known alleles at each locus by homology searching, with novel alleles identified and provided with a unique allele number. Loci with apparent truncations or sequencing errors in any of the genomes in the dataset were excluded from the analysis. The remaining loci (*n* = 732) were used to define the cgMLST scheme used in this study. The genetic distance between each pair of strains was calculated using the Hamming distance [59]. Hierarchical clustering was performed by the Unweighted Pair Group Method with Arithmetic Mean (UPGMA) using the *hclust* function in R [60].

## Additional files

Additional file 1: Figure S1. Dendrogram showing Neighbour-Joining analysis of *C. jejuni* complete whole genomes. The alignment was performed using Progressive Mauve [61] and the dendrogram produced using FigTree v1.4 [53]. (PDF 3 kb)
Additional file 2: Table S1. Selected SNVs detected using NUCmer for comparing all outbreak isolates using 00–2425 as reference. (DOCX 20 kb)
Additional file 3: Table S2. Homopolymeric G/C tracts eight nucleotides or longer in selected whole genome-sequenced *C. jejuni*. (DOCX 27 kb)
Additional file 4: Figure S2. Comparison of the ~93-kb invertible region for strains NCTC11168 = ATCC 700819 and 00–2425. Note that the 00–2425 region has been inverted for the diagram to highlight the synteny of this region between the two strains. The genes outside this region were found in the same locations in both NCTC11168 = ATCC 700819 and 00–2425, but appear at different places in the figure due to this inversion. Diagrams for each strain were obtained using GView Server [56] and further annotated using Adobe Illustrator. (PDF 177 kb)
Additional file 5: Table S3. Comparison of the ~93-kb inverted region of *C. jejuni* strains 00–2425 and NCTC11168. (DOCX 26 kb)
Additional file 6: Figure S3 Repeat sequences associated with isolates 00–2425, 00–2426, 00–2538, and 00–2544. (DOCX 22 kb)
Additional file 7: Figure S4. Isolate 00–2425 alignments of repeats associated with chemotaxis proteins. (DOCX 25 kb)

## Abbreviations

BIGSdb: Bacterial Isolate Genome Sequence Database
CC: Clonal complex
cgMLST: Core genome multilocus sequence typing
flaSVR: Flagellin A locus short variable region
HL: Heat-labile (serotype)
hqSNV: High quality single nucleotide variant
HS: Heat-stable (serotype)
MLST: Multilocus sequence typing
MOMP: Major outer membrane protein
nt: Nucleotide
PFGE: Pulsed-field gel electrophoresis
*porA* gene (MOMP): *porA* gene encoding the *C. jejuni* major outer membrane protein
rMLST: Ribosomal MLST
ST: *C. jejuni* MLST sequence type
Tlp: Transducer-like protein
WGS: Next-generation whole-genome sequencing

## References

[1] Coker AO, Isokpehi RD, Thomas BN, Amisu KO, Obi CL (2002). Human campylobacteriosis in developing countries. Emerg Infect Dis.

[2] Kirkpatrick BD, Tribble DR (2010). Update on human *Campylobacter jejuni* infections. Curr Opin Gastroenterol.

[3] Newell DG, Coopmans M, Verhoef L, Duizer E, Aidara-Kane A, Sprong H (2010). Food-borne diseases – the challenges of 20 years ago still persist while new ones continue to emerge. Int J Food Microbiol.

[4] Silva J, Leite D, Fernandes M, Mena C, Gibbs PA, Teixeria P (2011). Campylobacter spp. as a foodborne pathogen: a review. Frontiers Microbiol.

[5] Thomas MK, Murray R, Flockhart L, Pintar K, Pollari F, Fazil A (2013). Estimates of the burden of foodborne illness in Canada for 30 specified pathogens and unspecified agents, circa 2006. Foodborne Path Dis.

[6] Bronowski C, James CE, Winstanley C (2014). Role of environmental survival in transmission of *Campylobacter jejuni*. FEMS Microbiol Lett.

[7] Moore JE, Corcoran D, Dooley JSG, Fanning S, Lucey B, Matsuda M (2005). Campylobacter. Vet Res.

[8] Ahmed MU, Dunn L, Ivanova EP (2012). Evaluation of current molecular approaches for genotyping *Campylobacter jejuni* strains. Foodborne Path Dis.

[9] Taboada EN, Clark C, Sproston EL, Carrillo CD (2013). Current methods for molecular typing of *Campylobacter* species. J Microb Meth.

[10] Frost JA (2001). Current epidemiological issues in human campylobacteriosis. J Appl Micriobiol.

[11] Gilmour MW, Graham M, Reimer A, van Domselaar G (2013). Public health genomics and the new molecular epidemiology of bacterial pathogens. Public Health Genomics.

[12] Hasman H, Saputra D, Sicheritz-Pontin T, Lund O, Svendsen CA, Frimodt-Møller M (2014). Rapid whole-genome sequencing for detection and characterization of microorganisms directly from clinical samples. J Clin Microbiol.

[13] Robinson ER, Walker TM, Pallen MJ (2013). Genomics and outbreak investigation: from sequence to consequence. Genome Med.

[14] Wilson DJ (2012). Insights from genomics into bacterial pathogen populations. PLoS Pathog.

[15] Taboada EN, Ross SL, Mutschall SK, Mackinnon JM, Roberts MJ, Buchanan CJ (2012). Development and validation of a comparative genomic fingerprinting method for high- resolution genotyping of *Campylobacter jejuni*. J Clin Microbiol.

[16] Méric G, Yahara K, Mageiros L, Pascoe B, Maiden MCJ, Jolley KA (2014). A reference pan-genome approach to comparative bacterial genomics: identification of novel epidemiological markers in pathogenic *Campylobacter*. PLoS One.

[17] Revez J, Zhang J, Schott T, Kivistö R, Rossi M, Hänninen M-L (2014). Genomic variation between *Campylobacter jejuni* isolates associated with milk-borne-disease outbreaks. J Clin Microbiol.

[18] Revez J, Llarene A-K, Schott T, Kuusi M, Hakkinen M, Kivistö R (2014). Genome analysis of *Campylobacter jejuni* strains from a waterborne outbreak. BMC Genomics.

[19] Kovanen SM, Kivistö RI, Rossi M, Schott T, Kärkkäinen U-M, Tuuminen T (2014). Multilocus sequence typing (MLST) and whole-genome MLST of *Campylobacter jejuni* isolates from human infections in three districts during a seasonal peak in Finland. J Clin Microbiol.

[20] Cody AJ, McCarthy ND, van Rensburg MJ, Isinkaye T, Bentley SD, Parkhill J (2013). Real-time genomic epidemiological evaluation of human *Campylobacter* isolates by use of whole-genome multilocus sequence typing. J Clin Microbiol.

[21] Achaz G, Coissac E, Netter P, Rocha EPC (2003). Associations between inverted repeats and the structural evolution of bacterial genomes. Genetics.

[22] Fouts DE, Mongodin EF, Mandrell RE, Miller WG, Rasko DA, Ravel J (2005). Major structural differences and novel potential virulence mechanisms from the genomes of multiple *Campylobacter* species. PLoS Biol.

[23] Morley L, McNally A, Paszkiewicz K, Corander J, Méric G, Sheppard SK (2015). Gene loss and lineage-specific restriction-modification systems associated with niche differentiation in the *Campylobacter jejuni* sequence type 403 clonal complex. Appl Environ Microbiol.

[24] Relman DA (2011). Microbial genomics and infectious disease. New Engl J Med.

[25] Bruce-Grey-Owen Sound Health Unit (2000). Waterborne outbreak of gastroenteritis associated with a contaminated municipal water supply, Walkerton, Ontario, May-June 2000. Can Comm Dis Rep.

[26] Clark CG, Bryden L, Cuff W, Johnson PL, Jamieson F, Ciebin B (2005). Use of the Oxford multilocus sequence typing protocol and sequencing of the flagellin short variable region to characterize isolates from a large outbreak of waterborne *Campylobacter* sp. strains in Walkerton, Ontario, Canada. J Clin Microbiol.

[27] Barton C, Ng L-K, Tyler SD, Clark CG (2007). Temperate bacteriophages affect pulsed-field gel electrophoresis patterns of *Campylobacter jejuni*. J Clin Microbiol.

[28] Clark CG, Grant CCR, Pollari F, Marshall B, Moses J, Tracz DM (2012). Effects of the *Campylobacter jejuni* CJIE1 prophage homologs on adherence and invasion in culture, patient symptoms, and source of infection. BMC Microbiol.

[29] Clark CG, Chong PM, McCorrister SJ, Simon P, Walker M, Lee DM (2014). The CJIE1 prophage of *Campylobacter jejuni* affects protein expression in growth media with and without bile salts. BMC Microbiol.

[30] Kurtz S, Phillipy A, Delcher AL, Smoot M, Shumway M, Antonescu C (2004). Versatile and open software for comparing large genomes. Genome Biol.

[31] The MUMmer 3 Manual. http://mummer.sourceforge.net/manual/.

[32] Jolley KA, Maiden MCJ (2010). BIGSdb: Scalable analysis of bacterial genome variation at the population level. BMC Bioinformatics.

[33] Aidley J, Bayliss CD, Sheppard SK, Méric G (2014). Repetitive DNA: a major source of genetic diversity in *Campylobacter* populations?. *Campylobacter* ecology and evolution.

[34] He Y, Yan X, Reed S, Xie Y, Chen CY, Irwin P (2015). Complete genome sequence of *Campylobacter jejuni* YH001 from beef liver, which contains a novel plasmid. Genome Announc.

[35] Marchant J, Wren B, Ketley J (2002). Exploiting genome sequence: predictions for mechanisms of *Campylobacter* chemotaxis. TRENDS Microbiol.

[36] Clark CG, Price L, Ahmed R, Woodward DL, Melito PL, Rodgers FG (2003). Characterization of waterborne outbreak-associated *Campylobacter jejuni*, Walkerton, Ontario. Emerg Infect Dis.

[37] Thomas DK, Lone AG, Selinger LB, Taboada EN, Uwiera RRE, Abbott DW (2014). Comparative variation within the genome of *Campylobacter jejuni* NCTC 11168 in human and murine hosts. PLoS One.

[38] Jerome JP, Bell JA, Plovanich-Jones AE, Barrick JE, Brown CT, Mansfield LS (2011). Standing genetic variation in contingency loci drives the rapid adaptation of *Campylobacter jejuni* to a novel host. PLoS One.

[39] Kim J-S, Artymovich KA, Hall DF, Smith EJ, Fulton R, Bell J (2012). Passage of *Campylobacter jejuni* through the chicken reservoir or mice promotes phase variation in contingency genes *Cj0045* and *Cj0170* that strongly associates with colonization and disease in a mouse model. Microbiol.

[40] Revez J, Zhang J, Schott T, Llarina A-K, Rossi M, Hänninen M-L (2013). Genetic heterogeneity of *Campylobacter jejuni* NCTC 11168 upon human infection. Infect Genet Evol.

[41] Alm RA, Ling L-SL, Moir DT, King BL, Brown ED, Doig PC (1999). Genomic-sequence comparison of two unrelated isolates of the human gastric pathogen *Helicobacter pylori*. Nature.

[42] Scott AE, Timms AR, Connerton PL, Loc Carrillo C, Radzum KA, Connerton IF (2007). Genome dynamics of *Campylobacter jejuni* in response to bacteriophage predation. PLoS Pathog.

[43] Kothapalli S, Nair S, Alokam S, Pang T, Khakhria R, Woodward D (2005). Diversity of genome structure in *Salmonella enterica* serovar Typhi populations. J Bacteriol.

[44] Gripp E, Hlahla D, Didelot X, Kops F, Maurischat S, Tedin K (2011). Closely related *Campylobacter jejuni* strains from different sources reveal a generalist rather than a specialist lifestyle. BMC Genomics.

[45] Rocha EPC, Danchin A, Viari A (1999). Functional and evolutionary roles of long repeats in prokaryotes. Res Microbiol.

[46] Bankevich A, Nurk S, Antipov D, Gurevich AA, Dvorkin M, Kulikov AS (2012). SPAdes: a new genome assembly algorithm and its application to single-cell sequencing. J Comput Biol.

[47] WombacShred. http://www.vicbioinformatics.com/software.wombac.shtml.

[48] PHAC-NML SNVPhyl (hqSNV) pipeline. https://github.com/phac-nml/snvphyl-galaxy.

[49] FreeBayes. https://github.com/ekg/freebayes.

[50] Garrison E, Marth G. Haplotype-based variant detection from short-read sequencing. arXiv preprint arXiv 2012;1207.3907 [q-bio.GN]

[51] Li H, Handsaker B, Wysoker A, Fennel T, Buan J, Homer N (2012). The sequence alignment/map (SAM) format and SAMtools. Bioinformatics.

[52] Guindon S, Dufayard JF, Lefort V, Anisimova N, Hordijk W, Gascuel O (2010). New algorithms and methods to estimate Maximum-Likelihood phylogenies: assessing the performance of PhyML 3.0.. System Biol.

[53] FigTree v1.4. http://tree.bio.ed.ac.uk/software/figtree/.

[54] Inouye M, Dashnow H, Raven L-A, Schultz MB, Pope BJ, Tomita T (2014). Rapid genomic surveillance for public health and hospital microbiology labs. Genome Med.

[55] Petkau A, Stuart-Edwards M, Stothard P, Van Domselaar G (2010). Interactive microbial visualization with GView. Bioinformatics.

[56] GView Server. https://server.gview.ca. Accessed 23 Mar 2016.

[57] Sheppard SK, Jolley KA, Maiden MCJ (2012). A gene-by-gene approach to bacterial population genomics: whole genome MLST of *Campylobacter*. Genes.

[58] Kruczkiewicz P, Mutschall S, Barker D, Thomas J, Van Domselaar G, Gannon VPJ, et al. MIST: a tool for rapid in silico generation of molecular data from bacterial genome sequences. Proc. Bioinforma. 2013 4th Int. Conf. Bioinforma. Models Methods Algorithms: 316–23

[59] Hamming RW (1950). Error detecting and error correcting codes. Bell Syst Tech J.

[60] Core Team R (2012). R: A language and environment for statistical computing.

[61] Darling AC, Mau B, Blattner FR, Perna MT. Mauve: multiple alignment of conserved genomes sequences with rearrangements. Genome Res. 2004;14:1394–403. http://darlinglab.org/mauve. Accessed 11 Jan 2016. 10.1101/gr.2289704 PMC442156 15231754

