# Supplementary figures and images for: Genomic insights from whole genome sequencing of four clonal outbreak Campylobacter jejuni assessed within the global C. jejuni population
Source: BMC Genomics. 2016 Dec 3;17:990. doi: 10.1186/s12864-016-3340-8 (PMC5135748; doi:10.1186/s12864-016-3340-8)

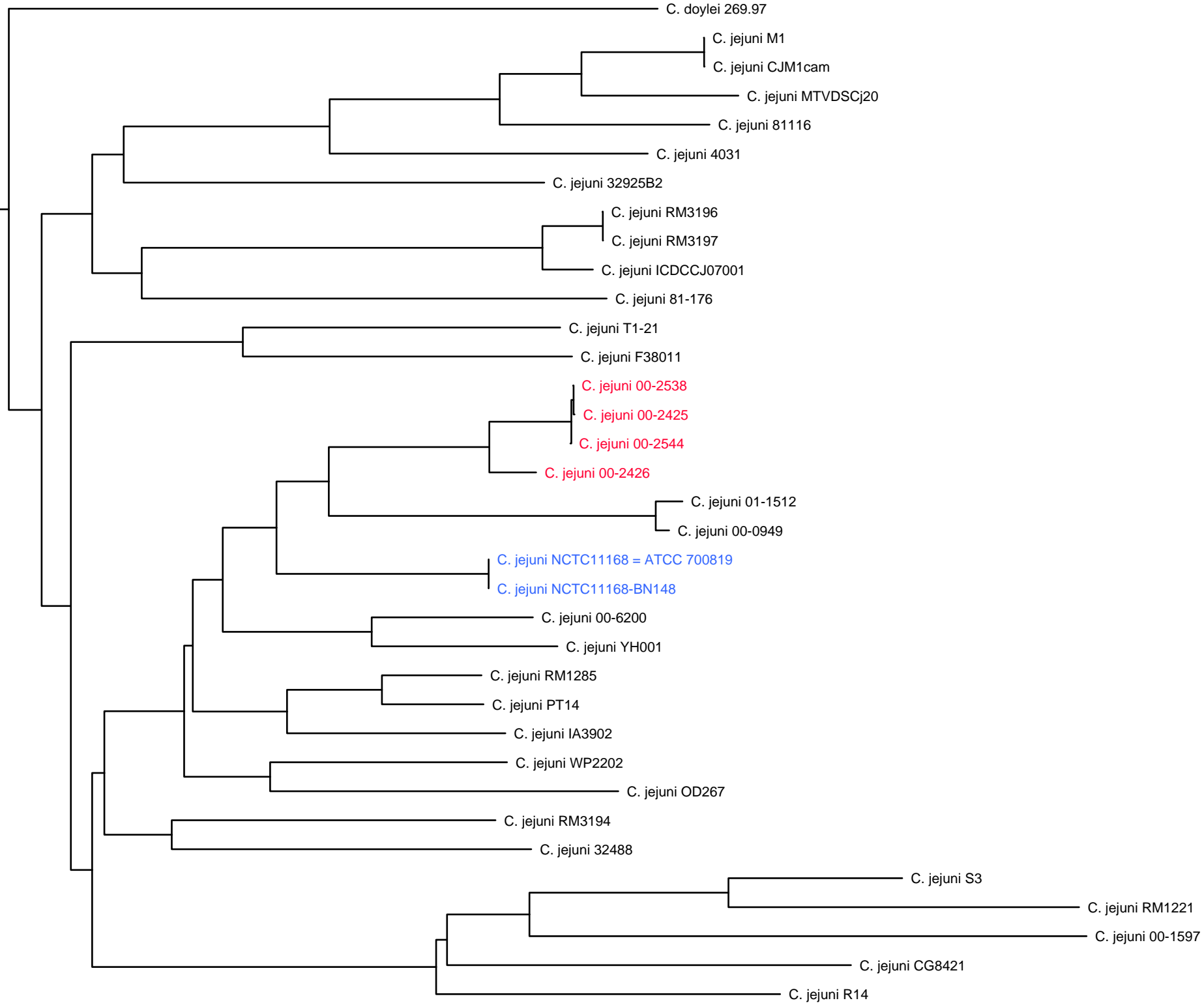

0.01

Supplement: Additional file 1: Figure S1. — Dendrogram showing Neighbour-Joining analysis of C. jejuni complete whole genomes. The alignment was performed using Progressive Mauve [61] and the dendrogram produced using FigTree v1.4 [53]. (PDF 3 kb) [file 12864_2016_3340_MOESM1_ESM.pdf]
