# Supplementary material for: Genomic insights from whole genome sequencing of four clonal outbreak Campylobacter jejuni assessed within the global C. jejuni population
Source: BMC Genomics. 2016 Dec 3;17:990. doi: 10.1186/s12864-016-3340-8 (PMC5135748; doi:10.1186/s12864-016-3340-8)
Supplement: Additional file 2: Table S1. — Selected SNVs detected using NUCmer for comparing all outbreak isolates using 00–2425 as reference. (DOCX 20 kb) [file 12864_2016_3340_MOESM2_ESM.docx]

**Additional file 2**

Table S1. Selected SNVs detected using NUCmer for comparing all outbreak isolates using 00-2425 as reference.

| Position | 00-2425 | 00-2426 | 00-2538 | 00-2544 | Protein | ATCC11168 locus |
| --- | --- | --- | --- | --- | --- | --- |
| 156251 | T | T | T | C | MFS transporter | Cj0250c |
| 194944 | T | T | T | C | amidophosphoribosyltransferase | Cj0196c |
| 531013 | T | T | T | C | hypothetical protein/periplasmic protein | Cj0530 |
| 549166 | . | . | . | G | flagellar protein FlaG | Cj0546 |
| 563358 | C | T | C | C | hypothetical protein | Cj0561c |
| 682886 | T | . | T | T | phosphate acetyltransferase | Cj0688 |
| 727719 | . | . | . | G | ABC transporter ATP-binding protein | Cj0616 |
| 960131 | T | T | T | . | MFS transporter | not annotated |
| 999533 | G | G | G | A | DNA gyrase subunit A | Cj1027c |
| 1073187 | T | G | T | T | serine/threonine transporter SstT | Cj1097 |
| 1180252 | C | C | C | T | 5-formyltetrahydrofolate cyclo-ligase | Cj1208 |
| 1216148 | T | T | T | C | hypothetical protein/membrane protein | Cj1245c |
| 1224808 | A | A | A | G | LPS-assembly protein | Cj1252 |
| 1232628 | A | A | A | G | major outer membrane protein | Cj1259 |
| 1346022 | T | T | T | C | flagellin B | Cj1338c |
| 1347856 | T | T | T | G | flagellin A | Cj1339c |
| 1347875 | G | G | G | A | flagellin A | Cj1339c |
| 1347880 | T | T | T | A | flagellin A | Cj1339c |
| 1347884 | A | A | A | G | flagellin A | Cj1339c |
| 1347886 | T | T | T | C | flagellin A | Cj1339c |
| 1347956 | T | T | T | G | flagellin A | Cj1339c |
| 1347961 | T | T | T | G | flagellin A | Cj1339c |
| 1347969 | T | T | T | C | flagellin A | Cj1339c |
| 1347977 | G | G | G | A | flagellin A | Cj1339c |
| 1347983 | A | A | A | T | flagellin A | Cj1339c |
| 1347986 | T | T | T | A | flagellin A | Cj1339c |
| 1347987 | G | G | G | A | flagellin A | Cj1339c |
| 1347992 | A | A | A | G | flagellin A | Cj1339c |
| 1348025 | C |  |  | A | flagellin A | Cj1339c |
| 1348028 | A |  |  | C | flagellin A | Cj1339c |
| 1351180 | G |  |  | A | motility accessory factor | Cj1341c |
| 1351182 | T |  |  | G | motility accessory factor | Cj1341c |
| 1351213 | A |  |  | G | motility accessory factor | Cj1341c |
| 1351225 | T |  |  | C | motility accessory factor | Cj1341c |
| 1351263 | C |  |  | T | motility accessory factor | Cj1341c |
| 1351282 | T |  |  | C | motility accessory factor | Cj1341c |
| 1351291 | G |  |  | A | motility accessory factor | Cj1341c |
| 1351294 | G |  |  | A | motility accessory factor | Cj1341c |
| 1351309 | T |  |  | C | motility accessory factor | Cj1341c |
| 1351324 | G |  |  | A | motility accessory factor | Cj1341c |
| 1351351 | G |  |  | A | motility accessory factor | Cj1341c |
| 1351360 | G |  |  | A | motility accessory factor | Cj1341c |
| 1351366 | G |  |  | A | motility accessory factor | Cj1341c |
| 1375689 | A |  |  | G | serine protease | Cj1365c |
| 1409637 | . |  |  | A | iron transporter FeoB – pseudogene | Cj1398 |
| 1409753 | . |  |  | T | iron transporter FeoB – pseudogene | Cj1398 |
| 1593438 | A |  |  | C | oxidoreductase/lactate dehydrogenase | Cj1585C |
| 1631572 | . |  |  | A | amino acid transporter; disrupted in 00-2544 | Cj1625c |
| 1707993 | C |  |  | T | 2-isopropylmalate synthase | Cj1719c |
