# Supplementary material for: Genomic insights from whole genome sequencing of four clonal outbreak Campylobacter jejuni assessed within the global C. jejuni population
Source: BMC Genomics. 2016 Dec 3;17:990. doi: 10.1186/s12864-016-3340-8 (PMC5135748; doi:10.1186/s12864-016-3340-8)
Supplement: Additional file 3: Table S2. — Homopolymeric G/C tracts eight nucleotides or longer in selected whole genome-sequenced C. jejuni. (DOCX 27 kb) [file 12864_2016_3340_MOESM3_ESM.docx]

**Additional file 3**

Table S2. Length of homopolymeric G/C tracts 8 nucleotides or longer in whole genome-sequenced *C. jejuni*.

| **Location (homolog)** | **YH001** | **NCTC 11168** | **PT14** | **RM 1221** | **S3** | **M1** | **ICDCC J07001** | **81-176** |
| --- | --- | --- | --- | --- | --- | --- | --- | --- |
| **upstream of L-asparaginase precursor Cj0029** | 10 | - | 10 | 10 | 10 | - | 10 | - |
| In Cj0031 type IIS restriction/modification enzyme | - | 10 | - | - | 9 | - | - | - |
| **In Cj0045c iron-binding protein** | 11 | 10 | 10 | 10 | 10 | 10 | 10 | 9 |
| In Cj0046 hypothetical protein | - | 11 | - | - | - | - | - | 9 |
| **In Cj0170 methylase/methyltransferase** | 11 | 9 | 9 | - | - | - | - | 9 |
| In Cj0275 (*clpX*) | 8 | - | - | - | - | - | - | - |
| Between Cj0275 (*clpX*) and Cj0276 (*mreB*) | - | 8 | - | - | - | - | - | - |
| Downstream of Cj0431 ATP/GTP-binding protein | - | - | - | - | - | - | 11 | - |
| **Downstream of Cj0564 (integral membrane protein)/upstream of Cj0565 (pseudogene in NCTC11168)** | - | 11 | 10 | 11 | 10 | 11 | 10 | 10 |
| **In Cj0617 carbonic anhydrase** | 9 | 9 | 10 | 9 | 9 | - | 10 | 9 |
| **In Cj0628-Cj0629 lipoprotein (10 G merges the two genes)** | - | 10 | 10 | - | - | - | - | - |
| **In kdpA potassium-transporting ATPase A subunit (Cj0674**  **pseudogene in NCTC11168)** | - | 9 | 10 | 10 | 9 | - | - | - |
| In kdpA potassium-transporting ATPase A subunit (Cj0675  pseudogene in NCTC11168) | - | - | - | 10 | 10 | - | - | - |
| **In Cj0685c invasion protein CipA** | 10 | 9 | 9 | 11 | 9 | 9 | 9 | 9 |
| In Cj0735 periplasmic protein | - | - | - | 13 | 10 | 9 | - | 9 |
| In ICDCCJ07001_711 adhesive protein CupB5 | - | - | - | - | - | - | 10 | - |
| **Between Cj0742 (membrane protein) and 16S rRNA locus; between ICDCCJ07001_713 and 16S locus** | 11 | 10 | 11 | 10 | 10 | 9 | 8 | 11 |
| In CJE1105 | - | - | - | 10 | - | - | - | - |
| **In Cj1051c *cjeI*, type I restriction modification enzyme** | 9 | - | - | - | - | - | 9 | - |
| CJM1_1115 rmlB | - | - | - | - | - | 9 | - | - |
| **In Cj1139c *wlaN*, *β*-1,3-galactosyltransferase** | - | 8 | 11 | - | 11 | - | - | - |
| In Cj1139c *wlaN,* *β*-1,3-galactosyltransferase, second site | - | - | - | - | 8 | - | - | - |
| In Cj1443? CMP-N-acetylneuraminic acid synthetase | 10 | - | - | - | - | - | - | 10 |
| In acetyltransferase QZ67_RS05960 (gene not in NCTC11168) | 10 | - | - | - | - | - | - | - |
| **In Cj1145 hypothetical protein** | - | 8 | - | 9 | - | - | - | - |
| In Cj1145 hypothetical protein, second site | - | - | - | 9 | - | - | - | - |
| Between CJS3_1300 a- CJS3_1301 | - | - | - | - | 8 | - | - | - |
| In Cj1255 tautomerase family protein | - | - | - | 11 | - | - | - | - |
| **In Cj1295 aminopeptidase** | 9 | 9 | 9 | 15 | 10 | 9 | - | 9 |
| **In Cj1296 pseudogene/AAC(3) family N-acetyltransferase** | 9 | 9 | 10 | - | - | - | - | - |
| **In Cj1305c hypothetical protein** | 9 | 9 | 9 | - | 9 | - | 9 | - |
| **In Cj1306c hypothetical protein** | 10 | 9 | 9 | 8 | - | - | - | 9 |
| In Cj1307 amino acid activating enzyme | - | - | - | 9 | 9 | - | - | 9 |
| In CJE1500 | - | - | - | 10 | 11 | - | - | - |
| Links CJE1502-1503 | - | - | - | 11 | 10 | - | - | - |
| In Cj1310c hypothetical protein | - | - | - | - | - | - | 9 | - |
| **In Cj1310c hypothetical protein** | 9 | 9 | 9 | 9 | 8 | 9 | 9 | - |
| In Cj1313 pseH N-acetyltransferase | - | - | - | - | - | 9 | - | - |
| ICDCCJ07001_1256 inserted between Cj1311 a- Cj1312 | - | - | - | - | - | - | 9 | - |
| In duplication of Cj1306c | - | - | 9 | - | - | - | - | - |
| In Cj1318 maf1/hypothetical protein | 9 | 11 | - | - | - | - | 9 | - |
| In SAM-dependent methyltransferase QZ67_RS07095 | 9 | - | - | - | - | - | - | - |
| In pseudogene QZ67_RS07100 | 9 | - | - | - | - | - | - | - |
| **In Cj1321 promoter region** | - | 10 | 10 | - | - | - | - | - |
| **In Cj1325 methyltransferase** | 10 | 10 | 9 | 9 | 9 | - | - | - |
| **In Cj1335 maf4 motility accessory factor** | 9 | 9 | 9 | - | - | - | 9 | - |
| In Cj1340 maf5 or 6 | - | - | - | - | - | 8 | - | - |
| **In Cj1342c maf7/hypothetical protein/carbonic anhydrase** | 9 | 9 | 9 | 9 | 9 | - | 9 | 9 |
| In CJE1549 (where Cj1360 would be in NCTC11168) | - | - | - | 8 | - | - | - | - |
| Between CJE1553 and CJE1552 | - | - | - | - | 10 | - | - | - |
| In CJE1602 | - | - | - | 9 | 9 | - | - | - |
| In CJE1603 | - | - | - | 9 | 10 | - | - | - |
| Between Cj1413c and Cj1414c homologs | - | - | - | - | - | - | 11 | - |
| **In Cj1420c methyltransferase** | 9 | 9 | 9 | - | - | 9 | - | - |
| **In Cj1421c sugar transferase** | 9 | 9 | 9 | - | - | - | - | 10 |
| **In Cj1422 sugar transferase** | 9 | 9 | - | - | - | - | 10 | - |
| In Cj1422 sugar transferase, second site | - | - | - | - | - | - | 9 | - |
| In ORF in same position as Cj1424 | - | - | - | - | - | - | 11 | - |
| **In Cj1426c methyltransferase** | - | 10 | - | - | - | - | - | - |
| **In Cj1429 hypothetical protein** | - | 10 | - | - | - | - | See below | - |
| In hypothetical protein QZ67_RS07580 | 8 | - | - | - | - | - | - | - |
| In GDP-mannose 4,6 dehydratase QZ67_RS07605 | 10 | - | - | - | - | - | - | - |
| ICDCC where fucose synthetase is in same position as Cj1429 | - | See above | - | - | - | - | 9 | - |
| Between A911_06917 and A911_06918 | - | - | 9 | - | - | - | - | - |
| In A911_06918 | - | - | 9 | - | - | - | - | - |
| **In Cj1437c hypothetical protein** | - | 9 | - | - | - | - | - | - |
| In ICDCCJ07001_1365 dTDP-4-dehydrorhamnose 3,5-epimerase | - | - | - | - | - | - | 11 | - |
| In CJJ81176_1432 sugar transferase | - | - | - | - | - | - | - | - |
| In CJJ81176_1435 sugar transferase | - | - | - | - | - | - | - | 9 |
| In sugar transferase A911_07000 | - | - | 9 | - | - | - | - | - |
| Cj0628 or A911_08080 | - | - | 10 | - | - | - | - | - |
| In ICDCCJ07001_1596 major facilitator family protein | - | - | - | - | - | - | 11 | - |
| **Total number of homopolymeric tracts** | 26 | 29 | 26 | 24 | 24 | 11 | 23 | 14 |

“-“, no homopolymeric tract detected; bold, homopolymeric tract also detected in the four outbreak isolates sequenced in this study
