# Supplementary material for: Genomic insights from whole genome sequencing of four clonal outbreak Campylobacter jejuni assessed within the global C. jejuni population
Source: BMC Genomics. 2016 Dec 3;17:990. doi: 10.1186/s12864-016-3340-8 (PMC5135748; doi:10.1186/s12864-016-3340-8)

http://clovr.org/docs/clusters-of-orthologous-groups-cogs/

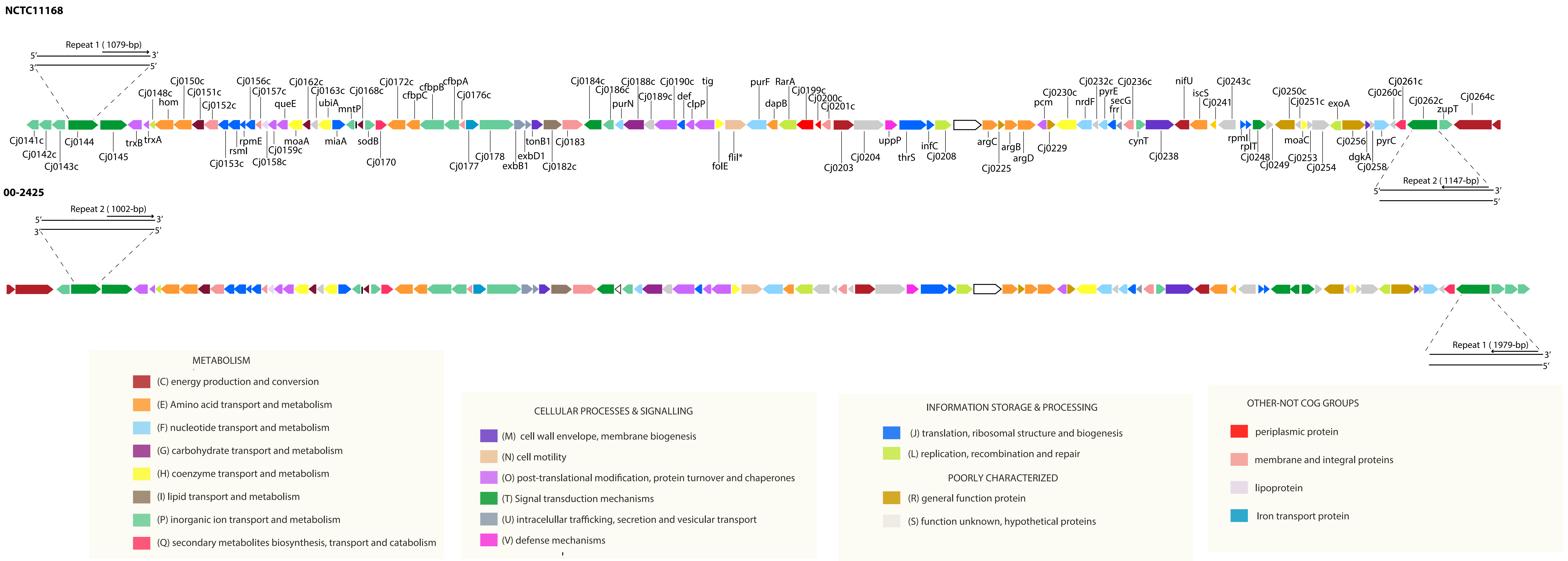

Supplement: Additional file 4: Figure S2. — Comparison of the ~93-kb invertible region for strains NCTC11168 = ATCC 700819 and 00–2425. Note that the 00–2425 region has been inverted for the diagram to highlight the synteny of this region between the two strains. The genes outside this region were found in the same locations in both NCTC11168 = ATCC 700819 and 00–2425, but appear at different places in the figure due to this inversion. Diagrams for each strain were obtained using GView Server [56] and further annotated using Adobe Illustrator. (PDF 177 kb) [file 12864_2016_3340_MOESM4_ESM.pdf]
