# Supplementary material for: Genomic insights from whole genome sequencing of four clonal outbreak Campylobacter jejuni assessed within the global C. jejuni population
Source: BMC Genomics. 2016 Dec 3;17:990. doi: 10.1186/s12864-016-3340-8 (PMC5135748; doi:10.1186/s12864-016-3340-8)
Supplement: Additional file 5: Table S3. — Comparison of the ~93-kb inverted region of C. jejuni strains 00–2425 and NCTC11168. (DOCX 26 kb) [file 12864_2016_3340_MOESM5_ESM.docx]

**Additional file 5**

Table S3. Comparison of the inverted regions of *C. jejuni* strains 00-2425 and NCTC11168. Regions were reverse-complemented for better comparison of the synteny between the chemotaxis proteins; entries in bold type were in the same location in both strains and were not part of the inverted segment.

| **Locus tag**  **00-2425** | **Protein description in 00-2425** | **Locus tag NCTC11168** | **Protein description NCTC11168** |
| --- | --- | --- | --- |
| **N135_RS01270** | **cytochrome c (homologous to Cj0265c)** | **Cj0141c** | **ABC transporter permease** |
| **N135_RS01265** | **trimethylamine N-oxide reductase I catalytic subunit (homologous to Cj0264c)** | **Cj0142c** | **ABC transporter ATP-binding protein** |
| **N135_RS01260** | **zinc transporter ZupT** | **Cj0143c** | **ABC transporter substrate binding protein** |
| **N135_RS01255** | **ribose and galactose chemoreceptor protein (novel Tlp?; position analogous to Cj0262c)** | **Cj0144** | **methyl-accepting chemotaxis signal transduction protein (Tlp2)** |
| N135_RS01250 | transcriptional initiation protein Tat | Cj0145 | TAT pathway signal sequence domain-containing protein |
| N135_RS01245 | thioredoxin-disulfide reductase | *trxB* | thioredoxin reductase |
| N135_RS01240 | thiol reductase thioredoxin | *trxA* | thioredoxin |
| N135_RS01235 | hypothetical protein (homologous to Cj0148) | Cj0148c | hypothetical protein |
| N135_RS01230 | homoserine dehydrogenase | *hom* | homoserine dehydrogenase |
| N135_RS01225 | aminotransferase | Cj0150c | aminotransferase |
| N135_RS01220 | hypothetical protein (homologous to Cj0151c) | Cj0151c | periplasmic protein |
| N135_RS01215 | membrane protein | Cj0152c | membrane protein |
| N135_RS01210 | RNA methyltransferase | Cj0153c | rRNA methylase |
| N135_RS01205 | rRNA (cytidine-2'-O-)-methyltransferase (homologous to Cj0154c) | Cj0154c/*rsmI* | rRNA small subunit methyltransferase 1 |
| N135_RS01200 | 50S ribosomal protein L31 | *rpmE* | 50S ribosomal protein L31 |
| N135_RS01195 | 16S rRNA (uracil(1498)-N(3))-methyltransferase (homologous to Cj0156c) | Cj0156c | rRNA small subunit methyltransferase E |
| N135_RS01190 | membrane protein (homologous to Cj0157) | Cj0157c | integral membrane protein |
| N135_RS01185 | cytochrome CBB3 (homologous to Cj0158c) | Cj0158c | heme-binding lipoprotein |
| N135_RS01180 | 6-carboxy-5,6,7,8-tetrahydropterin synthase (homologous to Cj0159c) | Cj0159c | 6-pyruvoyl-tetrahydropterin synthase |
| N135_RS01175 | 7-carboxy-7-deazaguanine synthase | Cj0160c/*queE* | radical SAM domain-containing protein |
| N135_RS01170 | cyclic pyranopterin monophosphate synthase | *moaA* | cyclic pyranopterin monophosphate synthase |
| N135_RS01165 | hypothetical protein (homologous to Cj0162c) | Cj0162c | periplasmic protein |
| N135_RS01160 | hypothetical protein (homologous to Cj0163c) | Cj0163c | hypothetical protein |
| N135_RS01155 | *ubiA* 4-hydroxybenzoate octaprenyltransferase | *ubiA* | 4-hydroxybenzoate octaprenyltransferase |
| N135_RS01150 | tRNA dimethylallyltransferase | *miaA* | tRNA dimethylallyltransferase |
| N135_RS01145 | membrane protein (homologous to Cj0167c) | Cj0167c/*mntP* | integral membrane protein |
| N135_RS01140 | tRNA-Glu | tRNAGlu | tRNA |
| N135_RS01135 | hypothetical protein (homologous to Cj0168c) | Cj0168c | periplasmic protein |
| N135_RS01130 | superoxide dismutase [Fe] | *sodB* | superoxide dismutase |
| N135_RS01125 | methyltransferase (homologous to Cj0170, missing 18 aa at N-terminus) | Cj0170 | hypothetical protein |
| N135_RS01120 | saccharopine dehydrogenase | Cj0172c | saccharopine dehydrogenase |
| N135_RS01115 | iron ABC transporter ATP-binding protein | *cfbpC* | iron-uptake ABC transporter ATP-binding protein |
| N135_RS01110 | iron ABC transporter permease | *cfbpB* | iron-uptake ABC transporter permease |
| N135_RS01105 | Fe(3+) ABC transporter substrate-binding protein (homologous to *cdbpA*) | *cdbpA* | iron-uptake ABC transporter substrate-binding protein |
| N135_RS01100 | hypothetical protein (homologous to Cj0176c) | Cj0176c | lipoprotein |
| N135_RS01095 | iron transporter (homologous to Cj0177) | Cj0177 | iron transport protein |
| N135_RS01090 | TonB-dependent receptor | Cj0178 | Ton-B-dependent outer membrane receptor |
| N135_RS01085 | biopolymer transporter ExbB | *exbB1* | biopolymer transport protein |
| N135_RS01080 | biopolymer transporter ExbD | *exbD1* | biopolymer transport protein |
| N135_RS01075 | biopolymer transporter TonB | *tonB1* | TonB transport protein |
| N135_RS01070 | transporter (homologous to Cj0182) | Cj0182c | transmembrane transport protein |
| N135_RS01065 | transporter (homologous to Cj0183) | Cj0183 | integral membrane protein |
| N135_RS01060 | serine/threonine protein phosphatase | Cj0184c | serine/threonine protein phosphatase |
| N135_RS01055 | PhnA domain protein | Cj0185c | doesn't exist/not annotated |
| N135_RS01050 | membrane protein (homologous to Cj0186c) | Cj0186c | TerC family integral membrane protein |
| N135_RS01045 | phosphoribosylglycinamide formyltransferase | *purN* | phosphoribosylglycinamide formyltransferase |
| N135_RS01040 | bifunctional ADP-dependent (S)-NAD(P)H-hydrate dehydratase/NAD(P)H-hydrate epimerase (homologous to Cj0188c) | Cj0188c | kinase |
| N135_RS01035 | hypothetical protein (homologous to Cj0189c) | Cj0189c | hypothetical protein |
| N135_RS01030 | Fis family transcriptional regulator (homologous to Cj0190c) | Cj0190c | hypothetical protein |
| N135_RS01025 | peptide deformylase | Cj0191c/*def* | peptide deformylase |
| N135_RS01020 | ATP-dependent Clp protease proteolytic subunit | *clpP* | ATP-dependent Clp protease proteolytic subunit |
| N135_RS01015 | trigger factor | *tig* | trigger factor |
| N135_RS01010 | GTP cyclohydrolase I | *folE* | GTP cyclohydrolase I |
| N135_RS01005 | flagellum-specific ATP synthase | *fliI* | flagellum-specific ATP synthase |
| N135_RS01000 | amidophosphoribosyltransferase | *purF* | amidophosphoribosyltransferase |
| N135_RS00995 | 4-hydroxy-tetrahydrodipicolinate reductase | *dapB* | 4-hydroxy-tetrahydrodipicolinate reductase |
| N135_RS00990 | ATPase AAA (homologous to Cj0198c) | Cj0198c | recombination factor protein, RarA |
| N135_RS00985 | hypothetical protein (homologous to Cj0199c) | Cj0199c | periplasmic protein |
| N135_RS00980 | hypothetical protein (homologous to Cj0200c) | Cj0200c | periplasmic protein |
| N135_RS00975 | membrane protein (homologous to Cj0201c) | Cj0201c | integral membrane protein |
| N135_RS00970 | hypothetical protein |  | not annotated, but ORF encoding protein is present |
| N135_RS00965 | citrate transporter | Cj0203 | citrate transporter |
| N135_RS00960 | oligopeptide transporter, OPT family | Cj0204 | oligopeptide transporter |
| N135_RS00955 | undecaprenyl-diphosphatase | *uppP* | undecaprenyl-diphosphatase |
| N135_RS00950 | threonine--tRNA ligase | *thrS* | threonine-tRNA ligase |
| N135_RS00945 | translation initiation factor IF-3 | *infC* | translation initiation factor IF-3 |
| N135_RS00940 | restriction endonuclease subunit M (homologous to Cj0208) | Cj0208 | DNA modification methylase |
| N135_RS00935 | hypothetical protein | present in NCTC11168 |  |
| N135_RS00930 | hypothetical protein | present in NCTC11168 |  |
| N135_RS00925 | Fis family transcriptional regulator | present in NCTC11168 |  |
| N135_RS00920 | pathogenicity protein | present in NCTC11168 |  |
| N135_RS00915 | serine protease | present in NCTC11168 |  |
|  | there is 100% DNA homology over the region matched between Cj0223 and N135_RS00915 to RS00940; the region is the same but annotated differently; likely a pseudogene in 00-2425 as well | Cj0223 – annotated as a pseudogene; encompasses the 5 previous ORFs | pseudogene |
| N135_RS00910 | N-acetyl-gamma-glutamyl-phosphate reductase | *argC* | N-acetyl-gamma-glutamyl-phosphate reductase |
| N135_RS00905 | acetyltransferase | Cj0225 | acetyltransferase |
| N135_RS00900 | acetylglutamate kinase | *argB* | acetylglutamate kinase |
| N135_RS00895 | acetylornithine aminotransferase | *argD* | acetylornithine aminotransferase |
| N135_RS00890 | protein-L-isoaspartate O-methyltransferase | *pcm* | protein-L-isoaspartate O-methyltransferase |
| N135_RS00885 | gamma carbonic anhydrase family protein (homologous to Cj0229) | Cj0229 | acetyltransferase |
| N135_RS00880 | nicotinate phosphoribosyltransferase | Cj0230c | nicotinate phophoribosyltransferase |
| N135_RS00875 | ribonucleotide-diphosphate reductase subunit beta (homologous to *nrdF*) | *nrdF* | ribonucleotide-diphosphate reductase subunit beta |
| N135_RS00870 | membrane protein (homologous to Cj0232c) | Cj0232c | integral membrane protein |
| N135_RS00865 | orotate phosphoribosyltransferase | *pyrE* | orotate phosphoribosyltransferase |
| N135_RS00860 | ribosome recycling factor | *frr* | ribosome recycling factor |
| N135_RS00855 | preprotein translocase subunit SecG | *secG* | protein translation subunit SecG |
| N135_RS00850 | membrane protein | Cj0236c | integral membrane protein |
| N135_RS00845 | carbonic anhydrase | *cynT* | carbionic anhydrase |
| N135_RS00840 | mechanosensitive ion channel protein | Cj0238 | mechanosensitive ion channel family protein |
| N135_RS00835 | iron-sulfur cluster assembly scaffold protein NifU | Cj0239c/*nifU* | nitrogen fixation protein NifU |
| N135_RS00830 | cysteine desulfurase | *iscS* | cysteine desulfurase |
| N135_RS00825 | hypothetical protein (homologous to Cj0241) | Cj0241 | bacteriohemerythrin |
| N135_RS00820 | hypothetical protein (homologous to Cj0243c) | Cj0243c | hypothetical protein |
| N135_RS00815 | 50S ribosomal protein L35 | *rpmI* | 50S ribosomal protein L35 |
| N135_RS00810 | 50S ribosomal protein L20 | *rplT* | 50S ribosomal protein L20 |
| N135_RS00805 | chemotaxis protein (not similar to Cj0248) |  |  |
| N135_RS00800 | pseudogene (chemotaxis protein) |  |  |
| N135_RS00795 | HDOD domain-containing protein (homologous to Cj0248) | Cj0248 | hypothetical protein |
| N135_RS00790 | cysteine permease (homologous to Cj0249) | Cj0249 | hypothetical protein |
| N135_RS00785 | MFS transporter | Cj0250c | MFS transport protein |
| N135_RS00780 | hypothetical protein (homologous to Cj0251c) | Cj0251c | highly acidic protein |
| N135_RS00775 | cyclic pyranopterin monophosphate synthase accessory protein | *moaC* | cyclic pyranopterin monophosphate synthase accessory protein |
| N135_RS00770 | hypothetical protein (homologous to Cj0253) | CJ0253 | hypothetical protein |
| N135_RS00765 | hypothetical protein (homologous to Cj0254) | Cj0254 | hypothetical protein |
| N135_RS00760 | exodeoxyribonuclease | *exoA* | exodeoxyribonuclease |
| N135_RS00755 | lipid A/FlgG phosphoethanolamine transferase EptC (homologous to Cj0256) | Cj0256 | sulfatase family protein |
| N135_RS00750 | diacylglycerol kinase | *dgkA* | diacylglycerol kinase |
| N135_RS00745 | ArsR family transcriptional regulator | Cj0258 | ArsR family transcriptional regulator |
| N135_RS00740 | dihydroorotase | *pyrC* | dihydroorotase |
| N135_RS00735 | hypothetical protein | Cj0260c | hypothetical protein |
| N135_RS00730 | SAM-dependent methyltransferase | Cj0261c | SAM-dependent methyltransferase |
| **N135_RS00725** | **methyl-accepting chemotaxis protein (Tlp3; position analogous to Cj0144)** | **Cj0262c** | **methyl-accepting chemotaxis signal transduction protein (Tlp4)** |
| **N135_RS00720** | **ABC transporter substrate-binding protein (homologous to Cj0143c)** | **Cj0263** | **zinc transporter ZupT** |
| **N135_RS00715** | **cation ABC transporter ATP-binding protein (homologous to Cj0142c)** | **Cj0264c** | **molybdopterin containing oxidoreductase** |
| **N135_RS00710** | **membrane protein (homologous to Cj0141c)** | **Cj0265c** | **cytochrome C-type heme-binding protein** |
