# Supplementary material for: Genomic insights from whole genome sequencing of four clonal outbreak Campylobacter jejuni assessed within the global C. jejuni population
Source: BMC Genomics. 2016 Dec 3;17:990. doi: 10.1186/s12864-016-3340-8 (PMC5135748; doi:10.1186/s12864-016-3340-8)
Supplement: Additional file 6: Figure S3 — Repeat sequences associated with isolates 00–2425, 00–2426, 00–2538, and 00–2544. (DOCX 22 kb) [file 12864_2016_3340_MOESM6_ESM.docx]

**Additional file 6**

**Figure S3. Repeat sequences associated with isolates 00-2425, 00-2426, 00-2538, and 00-2544**

Repeat 1. Methyl-accepting chemotaxis protein-associated repeat (1,979 nt) associated with protein N135_RS00725

ttaaaaggaaaataatgaatagtattaaaatcaaactttccctcattgcaaatttaattgcaatttttgccttaattgttctaggtattgtaagtttttattttacaaaaacctcactatatgaaagcactcttaaaaatcaaactgacctacttaaagtcacacaatctaccgttgaagatttccgttccacaaatcaatcttttactagagctttagaaaaagatatcgcgaacttaccttatcaatctttaatcactgaagaaaatattattaacaatgttggtccaatattgaaatattatcatcatagtataaatgcactaaatgtttatttaggtttaaacaatggaaaagttttacttagtcaaaaatctaatgatgcaaaaatgcctgaattacgtgatgatttagatataaagacaaaagattggtatcaagaagctttaaaaacaaatgatatttttgttacaccagcatatttagatacagttttaaaacaatatgtaataacgtattctaaagctatttataaagatggtaaaatcataggggtactgggtgtcgatataccatcagaagatttgcaaaatttagttgcaaaaacccctggaaatacttttttatttgatcaaaaaaataaaatatttgcagcaaccaataaagaattattaaatccatccattgatcattctcctgttctaaatgcatataaactcaatggtgataacaacttcttctcttataagttaaataatgaagaaagacttggagcttgtactaaagtctttgcttatacagcttgtattaccgaaagcgctgatattataaataaacctatttataaagctgcatttattcaagccattgttgtcattattgtagtagtatttagcgtcatcctcctttatttcatcgtatcaaaatacctctccccacttgcagctatccaaacaggtttaacttcattctttgattttatcaaccataaaacaaaaaatgtttctactatagaagtaaaaagcaatgatgaatttggacaaatctcaaatgctatcaatgaaaacattcttgctactaaaagaggcttagaacaagacaatcaagccgttaaagaatcagttcaaaccgtatcagttgtagaaggtggtaatttaacagcaagaattactgctaatccaagaaacccacagcttattgaacttaaaaatgttctaaataaacttcttgatgttttacaagctagagtaggttctgatatgaatgctattcataaaatttttgaagaatacaaaagcttagactttagaaataaattagaaaatgctagcggtagtgtagaattaactactaatgctttaggtgatgaaatagttaaaatgctaaaacaaagttcagactttgctaatgctttagctaatgaaagtggaaaattacaaactgctgttcaaagcttaaccacttcttcaaattctcaagctcaatctttagaagaaactgcagcagctttagaagagatcacttcttctatgcaaaatgtttcagttaaaactagtgatgttatcactcaatccgaagagattaaaatgttacaggtattataggtgatattgcagatcaaatcaatcttttagctttaaatgcagctattgaagcagctcgtgctggagaacatggtagaggctttgcagtggtagctgatgaagttagaaagttagctgaaagaactcaaaagtctttatctgaaattgaagctaatactaatttacttgttcaatctatcaatgatatggcagaaagtattaaagaacaaactgcaggtatcactcaaatcaatgatagcgtagctcaaattgatcaaactactaaagataatgttgaaattgctaatgaatcagctattatttctagtacagtaagtgatatagctaataatatcttagaagatgttaagaagaagaggttttaattaatcatt

Repeat 2. Ribose and galactose chemoreceptor protein-associated repeat (1011 nt) associated with protein N135_RS01255

aatgattaattaaaacctcttcttcttaacatcttctaagatattattagctatatcacttactgtactagaaataatagctgattcattagcaatttcaacattatctttagtagtttgatcaatttgagctacgctatcattgatttgagtgatacctgcagtttgttctttaatactttctgccatatcattgatagattgaacaagtaaattagtattagcttcaatttcagataaagacttttgagttctttcagctaactttctaacttcatcagctaccactgcaaagcctctaccatgttctccagcacgagctgcttcaatagctgcatttaaagctaaaagattgatttgatctgcaatatcacctataatacctgtaacatttttaatctcttcggattgagtgataacatcactagttttaactgaaacattttgcatagaagaagtgatctcttctaaagctgctgcagtttcttctaaagattgagcttgagaatttgaagaagtggttaagctttgaacagcagtttgtaattttccactttcat

tagctaaagcattagcaaagtctgaactttgttttagcattttaactatttcatcacctaaagcattagtagttaattctacactaccgctagcattttctaatttatttctaaagtctaagcttttgtattcttcaaaaattttatgaatagcattcatatcagaacctactctagcttgtaaaacatcaagaagtttatttagaacatttttaagttcaataagctgtgggtttcttggattagcagtaattcttgctgttaaattaccaccttctacaactgatacggtttgaactgattctttaacggcttgattgtcttgttctaagcctcttttagtagcaagaatgttttcattgataattttacccatttgccccaattcatcattgctttttacttctatagtagaaacattttttgttttatggttgataaagtcaaagaa

Repeat 2. Ribose and galactose chemoreceptor protein-associated repeat - reverse complement

ttctttgactttatcaaccataaaacaaaaaatgtttctactatagaagtaaaaagcaatgatgaattggggcaaatgggtaaaattatcaatgaaaacattcttgctactaaaagaggcttagaacaagacaatcaagccgttaaagaatcagttcaaaccgtatcagttgtagaaggtggtaatttaacagcaagaattactgctaatccaagaaacccacagcttattgaacttaaaaatgttctaaataaacttcttgatgttttacaagctagagtaggttctgatatgaatgctattcataaaatttttgaagaatacaaaagcttagactttagaaataaattagaaaatgctagcggtagtgtagaattaactactaatgctttaggtgatgaaatagttaaaatgctaaaacaaagttcagactttgctaatgctttagctaatgaaagtggaaaattacaaactgctgttcaaagcttaaccacttcttcaaattctcaagctcaatctttagaagaaactgcagcagctttagaagagatcacttcttc

tatgcaaaatgtttcagttaaaactagtgatgttatcactcaatccgaagagattaaaaatgttacaggtattataggtgatattgcagatcaaatcaatcttttagctttaaatgcagctattgaagcagctcgtgctggagaacatggtagaggctttgcagtggtagctgatgaagttagaaagttagctgaaagaactcaaaagtctttatctgaaattgaagctaatactaatttacttgttcaatctatcaatgatatggcagaaagtattaaagaacaaactgcaggtatcactcaaatcaatgatagcgtagctcaaattgatcaaactactaaagataatgttgaaattgctaatgaatcagctattatttctagtacagtaagtgatatagctaataatatcttagaagatgttaagaagaagaggttttaattaatcatt

Repeat 3. Methyl-accepting chemotaxis protein-associated repeat (1,970 nt) associated with protein N135_RS08200

ttaaaaggaaaataatgaatagtattaaaatcaaactttccctcattgcaaatttaattgcaatttttgccttaattgttctaggtattgtaagtttttattttacaaaaacctcactatatgaaagcactcttaaaaatcaaactgacctacttaaagtcacacaatctaccgttgaagatttccgttccacaaatcaatcttttactagagctttagaaaaagatatcgcgaacttaccttatcaatctttaatcactgaagaaaatattattaacaatgttggtccaatattgaaatattatcatcatagtataaatgcactaaatgtttatttaggtttaaacaatggaaaagttttacttagtcaaaaatctaatgatgcaaaaatgcctgaattacgtgatgatttagatataaagacaaaagattggtatcaagaagctttaaaaacaaatgatatttttgttacaccagcatatttagatacagttttaaaacaatatgtaataacgtattctaaagctatttataaagatggtaaaatcataggggtactgggtgtcgatataccatcagaagatttgcaaaatttagttgcaaaaacccctggaaatacttttttatttgatcaaaaaaataaaatatttgcagcaaccaataaagaattattaaatccatccattgatcattctcctgttctaaatgcatataaactcaatggtgataacaacttcttctcttataagttaaataatgaagaaagacttggagcttgtactaaagtctttgcttatacagcttgtattaccgaaagcgctgatattataaataaacctatttataaagctgcatttattcaagccattgttgtcattattgtagtagtatttagcgtcatcctcctttatttcatcgtatcaaaatacctctccccacttgcagctatccaaacaggtttaacttcattctttgattttatcaaccataaaacaaaaaatgtttctactatagaagtaaaaagcaatgatgaatttggacaaatctcaaatgctatcaatgaaaacattcttgctactaaaagaggcttagaacaagacaatcaagccgttaaagaatc

agttcaaaccgtatcagttgtagaaggtggtaatttaacagcaagaattactgctaatccaagaaacccacagcttattgaacttaaaaatgttctaaataaacttcttgatgttttacaagctagagtaggttctgatatgaatgctattcataaaatttttgaagaatacaaaagcttagactttagaaataaattagaaaatgctagcggtagtgtagaattaactactaatgctttaggtgatgaaatagttaaaatgctaaaacaaagttcagactttgctaatgctttagctaatgaaagtggaaaattacaaactgctgttcaaagcttaaccacttcttcaaattctcaagctcaatctttagaagaaactgcagcagctttagaagagatcacttcttctatgcaaaatgtttcagttaaaactagtgatgttatcactcaatccgaagagattaaaaatgttacaggtattataggtgatattgcagatcaaatcaatcttttagctttaaatgcagctattgaagcagctcgtgctggagaacatggta

gaggctttgcagtggtagctgatgaagttagaaagttagctgaaagaactcaaaagtctttatctgaaattgaagctaatactaatttacttgttcaatctatcaatgatatggcagaaagtattaaagaacaaactgcaggtatcactcaaatcaatgatagcgtagctcaaattgatcaaactactaaagataatgttgaaattgctaatgaatcagctattatttctagtacagtaagtgatatagctaataatatcttagaagatattaagaagaagaggttttaa
