# Supplementary material for: Genomic insights from whole genome sequencing of four clonal outbreak Campylobacter jejuni assessed within the global C. jejuni population
Source: BMC Genomics. 2016 Dec 3;17:990. doi: 10.1186/s12864-016-3340-8 (PMC5135748; doi:10.1186/s12864-016-3340-8)
Supplement: Additional file 7: Figure S4. — Isolate 00–2425 alignments of repeats associated with chemotaxis proteins. (DOCX 25 kb) [file 12864_2016_3340_MOESM7_ESM.docx]

**Additional File 7**

Figure S4. Strain 00-2425 alignments of repeats associated with chemotaxis proteins

A. Repeat 1 associated with the first chemotaxis gene encoding Tlp3 (N135_RS00725**;** 00-2425a) vs repeat 3 associated with the third chemotaxis gene encoding Tlp3 (N135_RS08200; 00-2425c).

**CLUSTAL O(1.2.1) multiple sequence alignment: done 2016/02/23**

00-2425a ttaaaaggaaaataatgaatagtattaaaatcaaactttccctcattgcaaatttaattg

00-2425c ttaaaaggaaaataatgaatagtattaaaatcaaactttccctcattgcaaatttaattg

************************************************************

00-2425a caatttttgccttaattgttctaggtattgtaagtttttattttacaaaaacctcactat

00-2425c caatttttgccttaattgttctaggtattgtaagtttttattttacaaaaacctcactat

************************************************************

00-2425a atgaaagcactcttaaaaatcaaactgacctacttaaagtcacacaatctaccgttgaag

00-2425c atgaaagcactcttaaaaatcaaactgacctacttaaagtcacacaatctaccgttgaag

************************************************************

00-2425a atttccgttccacaaatcaatcttttactagagctttagaaaaagatatcgcgaacttac

00-2425c atttccgttccacaaatcaatcttttactagagctttagaaaaagatatcgcgaacttac

************************************************************

00-2425a cttatcaatctttaatcactgaagaaaatattattaacaatgttggtccaatattgaaat

00-2425c cttatcaatctttaatcactgaagaaaatattattaacaatgttggtccaatattgaaat

************************************************************

00-2425a attatcatcatagtataaatgcactaaatgtttatttaggtttaaacaatggaaaagttt

00-2425c attatcatcatagtataaatgcactaaatgtttatttaggtttaaacaatggaaaagttt

************************************************************

00-2425a tacttagtcaaaaatctaatgatgcaaaaatgcctgaattacgtgatgatttagatataa

00-2425c tacttagtcaaaaatctaatgatgcaaaaatgcctgaattacgtgatgatttagatataa

************************************************************

00-2425a agacaaaagattggtatcaagaagctttaaaaacaaatgatatttttgttacaccagcat

00-2425c agacaaaagattggtatcaagaagctttaaaaacaaatgatatttttgttacaccagcat

************************************************************

00-2425a atttagatacagttttaaaacaatatgtaataacgtattctaaagctatttataaagatg

00-2425c atttagatacagttttaaaacaatatgtaataacgtattctaaagctatttataaagatg

************************************************************

00-2425a gtaaaatcataggggtactgggtgtcgatataccatcagaagatttgcaaaatttagttg

00-2425c gtaaaatcataggggtactgggtgtcgatataccatcagaagatttgcaaaatttagttg

************************************************************

00-2425a caaaaacccctggaaatacttttttatttgatcaaaaaaataaaatatttgcagcaacca

00-2425c caaaaacccctggaaatacttttttatttgatcaaaaaaataaaatatttgcagcaacca

************************************************************

00-2425a ataaagaattattaaatccatccattgatcattctcctgttctaaatgcatataaactca

00-2425c ataaagaattattaaatccatccattgatcattctcctgttctaaatgcatataaactca

************************************************************

00-2425a atggtgataacaacttcttctcttataagttaaataatgaagaaagacttggagcttgta

00-2425c atggtgataacaacttcttctcttataagttaaataatgaagaaagacttggagcttgta

************************************************************

00-2425a ctaaagtctttgcttatacagcttgtattaccgaaagcgctgatattataaataaaccta

00-2425c ctaaagtctttgcttatacagcttgtattaccgaaagcgctgatattataaataaaccta

************************************************************

00-2425a tttataaagctgcatttattcaagccattgttgtcattattgtagtagtatttagcgtca

00-2425c tttataaagctgcatttattcaagccattgttgtcattattgtagtagtatttagcgtca

************************************************************

00-2425a tcctcctttatttcatcgtatcaaaatacctctccccacttgcagctatccaaacaggtt

00-2425c tcctcctttatttcatcgtatcaaaatacctctccccacttgcagctatccaaacaggtt

************************************************************

00-2425a taacttcattctttgattttatcaaccataaaacaaaaaatgtttctactatagaagtaa

00-2425c taacttcattctttgattttatcaaccataaaacaaaaaatgtttctactatagaagtaa

************************************************************

00-2425a aaagcaatgatgaatttggacaaatctcaaatgctatcaatgaaaacattcttgctacta

00-2425c aaagcaatgatgaatttggacaaatctcaaatgctatcaatgaaaacattcttgctacta

************************************************************

00-2425a aaagaggcttagaacaagacaatcaagccgttaaagaatcagttcaaaccgtatcagttg

00-2425c aaagaggcttagaacaagacaatcaagccgttaaagaatcagttcaaaccgtatcagttg

************************************************************

00-2425a tagaaggtggtaatttaacagcaagaattactgctaatccaagaaacccacagcttattg

00-2425c tagaaggtggtaatttaacagcaagaattactgctaatccaagaaacccacagcttattg

************************************************************

00-2425a aacttaaaaatgttctaaataaacttcttgatgttttacaagctagagtaggttctgata

00-2425c aacttaaaaatgttctaaataaacttcttgatgttttacaagctagagtaggttctgata

************************************************************

00-2425a tgaatgctattcataaaatttttgaagaatacaaaagcttagactttagaaataaattag

00-2425c tgaatgctattcataaaatttttgaagaatacaaaagcttagactttagaaataaattag

************************************************************

00-2425a aaaatgctagcggtagtgtagaattaactactaatgctttaggtgatgaaatagttaaaa

00-2425c aaaatgctagcggtagtgtagaattaactactaatgctttaggtgatgaaatagttaaaa

************************************************************

00-2425a tgctaaaacaaagttcagactttgctaatgctttagctaatgaaagtggaaaattacaaa

00-2425c tgctaaaacaaagttcagactttgctaatgctttagctaatgaaagtggaaaattacaaa

************************************************************

00-2425a ctgctgttcaaagcttaaccacttcttcaaattctcaagctcaatctttagaagaaactg

00-2425c ctgctgttcaaagcttaaccacttcttcaaattctcaagctcaatctttagaagaaactg

************************************************************

00-2425a cagcagctttagaagagatcacttcttctatgcaaaatgtttcagttaaaactagtgatg

00-2425c cagcagctttagaagagatcacttcttctatgcaaaatgtttcagttaaaactagtgatg

************************************************************

00-2425a ttatcactcaatccgaagagatta-aaatgttacaggtattataggtgatattgcagatc

00-2425c ttatcactcaatccgaagagattaaaaatgttacaggtattataggtgatattgcagatc

************************ ***********************************

00-2425a aaatcaatcttttagctttaaatgcagctattgaagcagctcgtgctggagaacatggta

00-2425c aaatcaatcttttagctttaaatgcagctattgaagcagctcgtgctggagaacatggta

************************************************************

00-2425a gaggctttgcagtggtagctgatgaagttagaaagttagctgaaagaactcaaaagtctt

00-2425c gaggctttgcagtggtagctgatgaagttagaaagttagctgaaagaactcaaaagtctt

************************************************************

00-2425a tatctgaaattgaagctaatactaatttacttgttcaatctatcaatgatatggcagaaa

00-2425c tatctgaaattgaagctaatactaatttacttgttcaatctatcaatgatatggcagaaa

************************************************************

00-2425a gtattaaagaacaaactgcaggtatcactcaaatcaatgatagcgtagctcaaattgatc

00-2425c gtattaaagaacaaactgcaggtatcactcaaatcaatgatagcgtagctcaaattgatc

************************************************************

00-2425a aaactactaaagataatgttgaaattgctaatgaatcagctattatttctagtacagtaa

00-2425c aaactactaaagataatgttgaaattgctaatgaatcagctattatttctagtacagtaa

************************************************************

00-2425a gtgatatagctaataatatcttagaagatgttaagaagaagaggttttaattaatcatt

00-2425c gtgatatagctaataatatcttagaagatattaagaagaagaggttttaa---------

*****************************.********************

Sequence identity 1969/1971 nucleotides = 99.9%; 1 gap and 1 substitution

B. Repeat 1 associated with first chemotaxis gene encoding Tlp3 (N135_RS00725**;** 00-2425a) vs repeat 2 associated with the second chemotaxis/chemoreceptor gene encoding Tlp12 (N135_RS01255; 00-2425b, reverse complemented)

**CLUSTAL O(1.2.1) multiple sequence alignment: done 2016/02/23**

00-2425a ttaaaaggaaaataatgaatagtattaaaatcaaactttccctcattgcaaatttaattg

00-2425b ------------------------------------------------------------

00-2425a caatttttgccttaattgttctaggtattgtaagtttttattttacaaaaacctcactat

00-2425b ------------------------------------------------------------

00-2425a atgaaagcactcttaaaaatcaaactgacctacttaaagtcacacaatctaccgttgaag

00-2425b ------------------------------------------------------------

00-2425a atttccgttccacaaatcaatcttttactagagctttagaaaaagatatcgcgaacttac

00-2425b ------------------------------------------------------------

00-2425a cttatcaatctttaatcactgaagaaaatattattaacaatgttggtccaatattgaaat

00-2425b ------------------------------------------------------------

00-2425a attatcatcatagtataaatgcactaaatgtttatttaggtttaaacaatggaaaagttt

00-2425b ------------------------------------------------------------

00-2425a tacttagtcaaaaatctaatgatgcaaaaatgcctgaattacgtgatgatttagatataa

00-2425b ------------------------------------------------------------

00-2425a agacaaaagattggtatcaagaagctttaaaaacaaatgatatttttgttacaccagcat

00-2425b ------------------------------------------------------------

00-2425a atttagatacagttttaaaacaatatgtaataacgtattctaaagctatttataaagatg

00-2425b ------------------------------------------------------------

00-2425a gtaaaatcataggggtactgggtgtcgatataccatcagaagatttgcaaaatttagttg

00-2425b ------------------------------------------------------------

00-2425a caaaaacccctggaaatacttttttatttgatcaaaaaaataaaatatttgcagcaacca

00-2425b ------------------------------------------------------------

00-2425a ataaagaattattaaatccatccattgatcattctcctgttctaaatgcatataaactca

00-2425b ------------------------------------------------------------

00-2425a atggtgataacaacttcttctcttataagttaaataatgaagaaagacttggagcttgta

00-2425b ------------------------------------------------------------

00-2425a ctaaagtctttgcttatacagcttgtattaccgaaagcgctgatattataaataaaccta

00-2425b ------------------------------------------------------------

00-2425a tttataaagctgcatttattcaagccattgttgtcattattgtagtagtatttagcgtca

00-2425b ------------------------------------------------------------

00-2425a tcctcctttatttcatcgtatcaaaatacctctccccacttgcagctatccaaacaggtt

00-2425b ------------------------------------------------------------

00-2425a taacttcattctttgattttatcaaccataaaacaaaaaatgtttctactatagaagtaa

00-2425b --------ttctttgactttatcaaccataaaacaaaaaatgtttctactatagaagtaa

******** *******************************************

00-2425a aaagcaatgatgaatttggacaaatctcaaatgctatcaatgaaaacattcttgctacta

00-2425b aaagcaatgatgaattggggcaaatgggtaaaattatcaatgaaaacattcttgctacta

**************** ** ***** ** **************************

00-2425a aaagaggcttagaacaagacaatcaagccgttaaagaatcagttcaaaccgtatcagttg

00-2425b aaagaggcttagaacaagacaatcaagccgttaaagaatcagttcaaaccgtatcagttg

************************************************************

00-2425a tagaaggtggtaatttaacagcaagaattactgctaatccaagaaacccacagcttattg

00-2425b tagaaggtggtaatttaacagcaagaattactgctaatccaagaaacccacagcttattg

************************************************************

00-2425a aacttaaaaatgttctaaataaacttcttgatgttttacaagctagagtaggttctgata

00-2425b aacttaaaaatgttctaaataaacttcttgatgttttacaagctagagtaggttctgata

************************************************************

00-2425a tgaatgctattcataaaatttttgaagaatacaaaagcttagactttagaaataaattag

00-2425b tgaatgctattcataaaatttttgaagaatacaaaagcttagactttagaaataaattag

************************************************************

00-2425a aaaatgctagcggtagtgtagaattaactactaatgctttaggtgatgaaatagttaaaa

00-2425b aaaatgctagcggtagtgtagaattaactactaatgctttaggtgatgaaatagttaaaa

************************************************************

00-2425a tgctaaaacaaagttcagactttgctaatgctttagctaatgaaagtggaaaattacaaa

00-2425b tgctaaaacaaagttcagactttgctaatgctttagctaatgaaagtggaaaattacaaa

************************************************************

00-2425a ctgctgttcaaagcttaaccacttcttcaaattctcaagctcaatctttagaagaaactg

00-2425b ctgctgttcaaagcttaaccacttcttcaaattctcaagctcaatctttagaagaaactg

************************************************************

00-2425a cagcagctttagaagagatcacttcttctatgcaaaatgtttcagttaaaactagtgatg

00-2425b cagcagctttagaagagatcacttcttctatgcaaaatgtttcagttaaaactagtgatg

************************************************************

00-2425a ttatcactcaatccgaagagatta-aaatgttacaggtattataggtgatattgcagatc

00-2425b ttatcactcaatccgaagagattaaaaatgttacaggtattataggtgatattgcagatc

************************ ***********************************

00-2425a aaatcaatcttttagctttaaatgcagctattgaagcagctcgtgctggagaacatggta

00-2425b aaatcaatcttttagctttaaatgcagctattgaagcagctcgtgctggagaacatggta

************************************************************

00-2425a gaggctttgcagtggtagctgatgaagttagaaagttagctgaaagaactcaaaagtctt

00-2425b gaggctttgcagtggtagctgatgaagttagaaagttagctgaaagaactcaaaagtctt

************************************************************

00-2425a tatctgaaattgaagctaatactaatttacttgttcaatctatcaatgatatggcagaaa

00-2425b tatctgaaattgaagctaatactaatttacttgttcaatctatcaatgatatggcagaaa

************************************************************

00-2425a gtattaaagaacaaactgcaggtatcactcaaatcaatgatagcgtagctcaaattgatc

00-2425b gtattaaagaacaaactgcaggtatcactcaaatcaatgatagcgtagctcaaattgatc

************************************************************

00-2425a aaactactaaagataatgttgaaattgctaatgaatcagctattatttctagtacagtaa

00-2425b aaactactaaagataatgttgaaattgctaatgaatcagctattatttctagtacagtaa

************************************************************

00-2425a gtgatatagctaataatatcttagaagatgttaagaagaagaggttttaattaatcatt

00-2425b gtgatatagctaataatatcttagaagatgttaagaagaagaggttttaattaatcatt

***********************************************************

Sequence identity of repeat 1001/1011 nucleotides = 99.0%; 1 gap and 10 substitutions

C. Repeat 2 associated with the second chemotaxis/chemoreceptor gene encoding Tlp12 (N135_RS01255; 00-2425b) vs repeat 3 associated with the third chemotaxis gene encoding Tlp3 (N135_RS08200; 00-2425c)

**CLUSTAL O(1.2.1) multiple sequence alignment: done 2016/02/23**

00-2425b ------------------------------------------------------------

00-2425c ttaaaaggaaaataatgaatagtattaaaatcaaactttccctcattgcaaatttaattg

00-2425b ------------------------------------------------------------

00-2425c caatttttgccttaattgttctaggtattgtaagtttttattttacaaaaacctcactat

00-2425b ------------------------------------------------------------

00-2425c atgaaagcactcttaaaaatcaaactgacctacttaaagtcacacaatctaccgttgaag

00-2425b ------------------------------------------------------------

00-2425c atttccgttccacaaatcaatcttttactagagctttagaaaaagatatcgcgaacttac

00-2425b ------------------------------------------------------------

00-2425c cttatcaatctttaatcactgaagaaaatattattaacaatgttggtccaatattgaaat

00-2425b ------------------------------------------------------------

00-2425c attatcatcatagtataaatgcactaaatgtttatttaggtttaaacaatggaaaagttt

00-2425b ------------------------------------------------------------

00-2425c tacttagtcaaaaatctaatgatgcaaaaatgcctgaattacgtgatgatttagatataa

00-2425b ------------------------------------------------------------

00-2425c agacaaaagattggtatcaagaagctttaaaaacaaatgatatttttgttacaccagcat

00-2425b ------------------------------------------------------------

00-2425c atttagatacagttttaaaacaatatgtaataacgtattctaaagctatttataaagatg

00-2425b ------------------------------------------------------------

00-2425c gtaaaatcataggggtactgggtgtcgatataccatcagaagatttgcaaaatttagttg

00-2425b ------------------------------------------------------------

00-2425c caaaaacccctggaaatacttttttatttgatcaaaaaaataaaatatttgcagcaacca

00-2425b ------------------------------------------------------------

00-2425c ataaagaattattaaatccatccattgatcattctcctgttctaaatgcatataaactca

00-2425b ------------------------------------------------------------

00-2425c atggtgataacaacttcttctcttataagttaaataatgaagaaagacttggagcttgta

00-2425b ------------------------------------------------------------

00-2425c ctaaagtctttgcttatacagcttgtattaccgaaagcgctgatattataaataaaccta

00-2425b ------------------------------------------------------------

00-2425c tttataaagctgcatttattcaagccattgttgtcattattgtagtagtatttagcgtca

00-2425b ------------------------------------------------------------

00-2425c tcctcctttatttcatcgtatcaaaatacctctccccacttgcagctatccaaacaggtt

00-2425b --------ttctttgactttatcaaccataaaacaaaaaatgtttctactatagaagtaa

00-2425c taacttcattctttgattttatcaaccataaaacaaaaaatgtttctactatagaagtaa

******** *******************************************

00-2425b aaagcaatgatgaattggggcaaatgggtaaaattatcaatgaaaacattcttgctacta

00-2425c aaagcaatgatgaatttggacaaatctcaaatgctatcaatgaaaacattcttgctacta

**************** **.***** :**:. **************************

00-2425b aaagaggcttagaacaagacaatcaagccgttaaagaatcagttcaaaccgtatcagttg

00-2425c aaagaggcttagaacaagacaatcaagccgttaaagaatcagttcaaaccgtatcagttg

************************************************************

00-2425b tagaaggtggtaatttaacagcaagaattactgctaatccaagaaacccacagcttattg

00-2425c tagaaggtggtaatttaacagcaagaattactgctaatccaagaaacccacagcttattg

************************************************************

00-2425b aacttaaaaatgttctaaataaacttcttgatgttttacaagctagagtaggttctgata

00-2425c aacttaaaaatgttctaaataaacttcttgatgttttacaagctagagtaggttctgata

************************************************************

00-2425b tgaatgctattcataaaatttttgaagaatacaaaagcttagactttagaaataaattag

00-2425c tgaatgctattcataaaatttttgaagaatacaaaagcttagactttagaaataaattag

************************************************************

00-2425b aaaatgctagcggtagtgtagaattaactactaatgctttaggtgatgaaatagttaaaa

00-2425c aaaatgctagcggtagtgtagaattaactactaatgctttaggtgatgaaatagttaaaa

************************************************************

00-2425b tgctaaaacaaagttcagactttgctaatgctttagctaatgaaagtggaaaattacaaa

00-2425c tgctaaaacaaagttcagactttgctaatgctttagctaatgaaagtggaaaattacaaa

************************************************************

00-2425b ctgctgttcaaagcttaaccacttcttcaaattctcaagctcaatctttagaagaaactg

00-2425c ctgctgttcaaagcttaaccacttcttcaaattctcaagctcaatctttagaagaaactg

************************************************************

00-2425b cagcagctttagaagagatcacttcttctatgcaaaatgtttcagttaaaactagtgatg

00-2425c cagcagctttagaagagatcacttcttctatgcaaaatgtttcagttaaaactagtgatg

************************************************************

00-2425b ttatcactcaatccgaagagattaaaaatgttacaggtattataggtgatattgcagatc

00-2425c ttatcactcaatccgaagagattaaaaatgttacaggtattataggtgatattgcagatc

************************************************************

00-2425b aaatcaatcttttagctttaaatgcagctattgaagcagctcgtgctggagaacatggta

00-2425c aaatcaatcttttagctttaaatgcagctattgaagcagctcgtgctggagaacatggta

************************************************************

00-2425b gaggctttgcagtggtagctgatgaagttagaaagttagctgaaagaactcaaaagtctt

00-2425c gaggctttgcagtggtagctgatgaagttagaaagttagctgaaagaactcaaaagtctt

************************************************************

00-2425b tatctgaaattgaagctaatactaatttacttgttcaatctatcaatgatatggcagaaa

00-2425c tatctgaaattgaagctaatactaatttacttgttcaatctatcaatgatatggcagaaa

************************************************************

00-2425b gtattaaagaacaaactgcaggtatcactcaaatcaatgatagcgtagctcaaattgatc

00-2425c gtattaaagaacaaactgcaggtatcactcaaatcaatgatagcgtagctcaaattgatc

************************************************************

00-2425b aaactactaaagataatgttgaaattgctaatgaatcagctattatttctagtacagtaa

00-2425c aaactactaaagataatgttgaaattgctaatgaatcagctattatttctagtacagtaa

************************************************************

00-2425b gtgatatagctaataatatcttagaagatgttaagaagaagaggttttaattaatcatt

00-2425c gtgatatagctaataatatcttagaagatattaagaagaagaggttttaa---------

*****************************.********************

Sequence identity of repeat 992/1003 nucleotides = 98.9%; no gaps and 11 substitutions
